# Revealing emergent magnetic charge in an antiferromagnet with diamond quantum magnetometry

**DOI:** 10.1038/s41563-023-01737-4

**Published:** 2023-12-05

**Authors:** Anthony K. C. Tan, Hariom Jani, Michael Högen, Lucio Stefan, Claudio Castelnovo, Daniel Braund, Alexandra Geim, Annika Mechnich, Matthew S. G. Feuer, Helena S. Knowles, Ariando Ariando, Paolo G. Radaelli, Mete Atatüre

**Affiliations:** 1 Cavendish Laboratory, University of Cambridge, Cambridge, UK; 2 Clarendon Laboratory, Department of Physics, University of Oxford, Oxford, UK; 3 Department of Physics, National University of Singapore, Singapore, Singapore; 4 Center for Hybrid Quantum Networks (Hy-Q), Niels Bohr Institute, University of Copenhagen, Copenhagen, Denmark

**Keywords:** Magnetic properties and materials, Spintronics, Condensed-matter physics, Imaging techniques, Topological defects

## Abstract

Whirling topological textures play a key role in exotic phases of magnetic materials and are promising for logic and memory applications. In antiferromagnets, these textures exhibit enhanced stability and faster dynamics with respect to their ferromagnetic counterparts, but they are also difficult to study due to their vanishing net magnetic moment. One technique that meets the demand of highly sensitive vectorial magnetic field sensing with negligible backaction is diamond quantum magnetometry. Here we show that an archetypal antiferromagnet—haematite—hosts a rich tapestry of monopolar, dipolar and quadrupolar emergent magnetic charge distributions. The direct read-out of the previously inaccessible vorticity of an antiferromagnetic spin texture provides the crucial connection to its magnetic charge through a duality relation. Our work defines a paradigmatic class of magnetic systems to explore two-dimensional monopolar physics, and highlights the transformative role that diamond quantum magnetometry could play in exploring emergent phenomena in quantum materials.

## Abstract (web summary)

Diamond quantum magnetometry is utilized to directly read the vorticity of antiferromagnetic spin textures through coupled multi-polar emergent magnetic charge distributions.

## Main

Topologically protected states in magnetic materials are promising candidates for next-generation spintronics architectures^1,2^. In particular, topological textures in antiferromagnets (AFMs) could provide additional advantages over their ferromagnetic counterparts including enhanced stability as well as faster and richer dynamics^3–17^. However, the vanishing net moment renders the detection of AFM textures difficult. Synchrotron-based dichroic X-ray techniques are at the imaging forefront and have—for the first time—revealed the existence of two-dimensional (2D) topological AFM spin textures in haematite, namely, α-Fe_2_O_3_ (refs. ^18,19^). Although sensitive to staggered magnetization, this technique is insensitive to its sign and thus the associated vorticity, that is, the whirling of the spin textures is not observed.

When viewed through the lens of canted magnetization, instead of the Néel vector, we uncover weak magnetic fields emanating from the divergence of the canted moments. Such fields can be equivalently described by the magnetic analogue of Gauss’s law^20^, thereby pointing to the existence of emergent magnetic charges in a topologically rich AFM landscape. Diamond quantum magnetometry (DQM), employing a single nitrogen-vacancy (NV) colour centre as a point field sensor, enables weak field sensing^21–25^, thereby putting it in a unique position to study the above proposed concept of emergent magnetic charges in a new class of magnetic materials—canted AFMs.

In this Article, we demonstrate the DQM imaging of topological textures in the AFM α-Fe_2_O_3_ and show that these textures host a rich tapestry of magnetic charge distribution. In particular, the duality relation between staggered vorticity and magnetic charge allows us to associate the AFM Bloch meron with a spatially extended emergent magnetic monopole. Distinct from emergent magnetic monopoles in other realizations, such as spin ice^26^, we observe that the positively and negatively charged monopolar textures are topologically equivalent, whereas the topological antiparticle (AFM antimeron) has a quadrupolar character. Our results demonstrate the potential of DQM to discover and investigate emergent magnetic phenomena.

## Properties of α-Fe_2_O_3_ and DQM

**Fig. 1:**
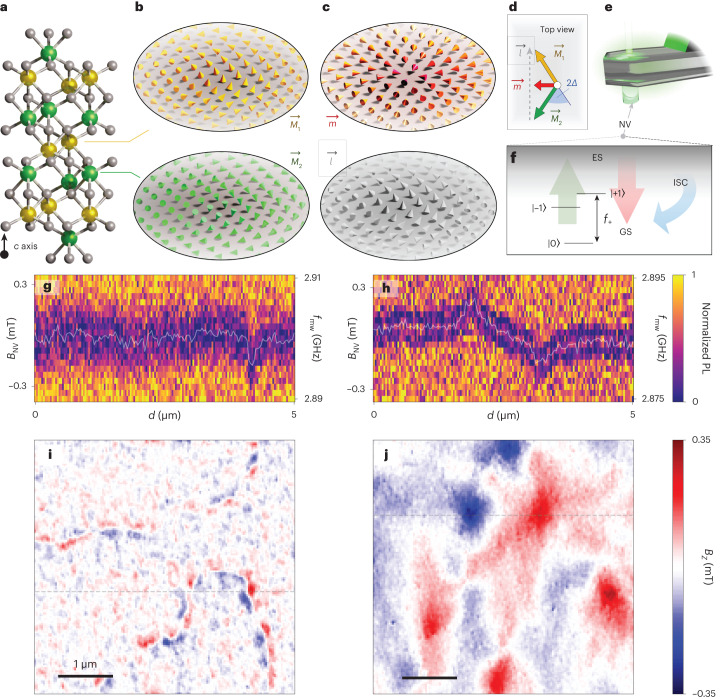
Signatures of emergent magnetic fields in α-Fe_2_O_3_. **a**, Atomic structure of α-Fe_2_O_3_ (Fe and O atoms in the yellow/green and grey spheres, respectively). **b**, Discrete representation of the alternating ferromagnetic sublattice magnetization $\overrightarrow{{M}_{1}}$ (yellow cones) and $\overrightarrow{{M}_{2}}$ (green cones) with AFM coupling along the *c* axis shown in **a**. **c**, Illustration of the whirling staggered magnetization $\overrightarrow{l}$ (grey cones), forming an anti-clockwise a-Bloch meron, and the resultant canted magnetic moment $\overrightarrow{m}$ (red cones). **d**, Illustration showing the relationship between $\overrightarrow{l}$, $\overrightarrow{m}$, $\overrightarrow{{M}_{1}}$ and $\overrightarrow{{M}_{2}}$ and the canting angle Δ. **e**, A scanning diamond sensor with a single NV centre maps out the magnetic ($\vec{B}$) field generated near the sample surface. **f**, Energy diagram of the NV ground states (GS) of |±1〉 and |0〉 sublevels. A microwave field drives the GS spin transition, whereas a 532 nm laser excites the NV to the excited state (ES) (green arrow). The NV then undergoes a radiative decay to GS (red arrow) or a non-radiative and spin-selective path via the intersystem crossing (ISC) (blue arrow), enabling ODMR acquisition. **g**,**h**, ODMR (mapped as the normalized photoluminescence (PL)) along the fast-scan direction, measured on the α-Fe_2_O_3_ thin film at *T* = 4 K (**g**) and across *T*_M_ at 300 K (**h**). The fitted *f*_+_(*B*_NV_) is plotted as a white line in each panel. **i**,**j**, *B*_*z*_ images retrieved from fitted *B*_NV_ maps reveal distinct field signatures across *T*_M_. The dashed lines in **i** and **j** correspond to fitted *B*_NV_ traces in **g** and **h**, respectively. Scale bars, 1 μm.

Haematite α-Fe_2_O_3_ is an AFM oxide insulator, which hosts a variety of topological spin textures^18,27,28^. Figure 1a illustrates the atomic structure of α-Fe_2_O_3_. It comprises a stack of anti-parallel ferromagnetic sublattices along the *c* axis, with magnetization textures $\overrightarrow{{M}_{1}}$ and $\overrightarrow{{M}_{2}}$ (Fig. 1b). Spin re-orientation occurs at the Morin transition temperature, *T*_M_ ≈ 200 K (ref. ^18^) (Supplementary Section 1); below and above *T*_M_, the ferromagnetic sublattices lie predominantly out of plane and in plane, respectively. The Néel vector $\overrightarrow{l}\,=\overrightarrow{{M}_{1}}-\overrightarrow{{M}_{2}}$ characterizes the AFM order, whereas $\overrightarrow{m}\,=\overrightarrow{{M}_{1}}+\overrightarrow{{M}_{2}}$ is the net magnetization (Fig. 1c,d). Above *T*_M_, $\overrightarrow{m}$ has a predominantly in-plane orientation with an average magnitude *m*_Δ_ ≈ 2 × 10^3^ A m^–1^. This weak magnetization is due to the slight in-plane canting of Δ ≈ 1.1 mrad (Supplementary Section 1) between $\overrightarrow{{M}_{1}}$ and $\overrightarrow{{M}_{2}}$, as a consequence of the bulk Dzyalonshinskii–Moriya interaction (DMI) vector along the *c* axis^29^ (Supplementary Section 5). Consequently, $\overrightarrow{m}$ lies in plane and satisfies $\overrightarrow{m}\cdot \overrightarrow{l}=0$. Since *m*_Δ_ is much weaker than $| \overrightarrow{l}|$, this weak magnetization has no discernible effect on the AFM character of α-Fe_2_O_3_. Finally, $\overrightarrow{m}$ vanishes where $\overrightarrow{l}$ turns out of plane below *T*_M_ or due to the formation of AFM spin textures.

We quantify the magnetic field distribution from these spin textures via DQM (Supplementary Section 2). Figure 1e illustrates our diamond probe hosting a single NV centre, which is scanned at a constant height above the sample. The NV centre is a spin defect with a paramagnetic ground-state manifold and state-selective optical transitions. This allows the Zeeman splitting between the ground states |±1〉 and |0〉 to be probed with a microwave frequency *f*_mw_ sweep and optical excitation via optically detected magnetic resonance (ODMR). In the weak-field approximation^30^ with negligible strain, we infer the magnetic field projected onto the NV axis (*B*_NV_) from the energy difference between |0〉 and |+1〉 given by ${\varDelta} E_{+}=h(|\;f_+-D| -{\tilde{\gamma }}{B}_{\rm{bias}})=h{\tilde{\gamma }}{B}_{\rm{NV}}$, where *h* is Planck’s constant, *f*_+_ is the resonant frequency corresponding to the transition, *D* ≈ 2.87 GHz and $\tilde{\gamma }$ = 28 MHz mT^–1^. A bias field *B*_bias_ ≈ 0.5 mT is applied along the NV axis to enable the extraction of field orientation. Figure 1g,h illustrates the variation in ODMR frequency across a linescan over the α-Fe_2_O_3_ surface at *T* = 4 and 300 K, respectively. The colour plot displays the signal amplitude, whereas the white curves demarcate *f*_+_ used to extract *B*_NV_. An ODMR raster scan across the sample surface provides a *B*_NV_ image. We transform this to the laboratory coordinates (*B*_*x*,*y*,*z*_) via the Fourier reconstruction technique^31^, where *z* coincides with the *c* axis of the crystal. Figure 1i,j presents images of *B*_*z*_ collected at 4 and 300 K, respectively. The observed qualitative differences reveal distinct magnetic phases for temperatures below and above *T*_M_ (ref. ^18^). The image below *T*_M_ comprises narrow features in an almost-zero field background, consistent with the absence of net magnetization. By contrast, the image above *T*_M_ displays larger features within a non-zero field background, expected from a non-zero net magnetization.

## Emergent properties in α-Fe_2_O_3_

To gain a physical interpretation of magnetization distribution from the measured *B*_*z*_ images, we begin from a thin-film approximation (Supplementary Section 4.1):

1 $${B}_{z}={\alpha }_{xy}(t,d)* \overrightarrow{\nabla }\cdot {\overrightarrow{m}}_{xy}+{\alpha }_{z}(t,d)* {\nabla }^{2}{m}_{z},$$

where * indicates convolution; *α*_*i*_ (*i* = *xy*, *z*) are the effective point spread functions^32^; *t* is the film thickness; *d* is the height above the film surface; and ${\overrightarrow{m}}_{xy}$ and *m*_*z*_ are the in-plane and out-of-plane components of $\overrightarrow{m}$, respectively. ${\overrightarrow{m}}_{xy}$ and *m*_*z*_ contribute to *B*_*z*_ through the divergence and Laplacian, respectively. The *α*_*i*_ functions account for the magnetic field decay above the surface, acting as blurring kernels with size of ~*d*. Hence, the spatial resolution of DQM is set by the NV-sample distance *d*_NV_ (Supplementary Section 3). Due to DMI symmetry in α-Fe_2_O_3_ (Supplementary Section 5), *m*_*z*_ = 0 and ${\overrightarrow{m}}_{xy}\ne 0$, rendering the second term in equation (1) zero. Therefore, *B*_*z*_ images are the divergence of the canted magnetization $\overrightarrow{\nabla }\cdot {\overrightarrow{m}}_{xy}$, convolved with *α*_*xy*_. Moreover, for a $\hat{z}$-oriented DMI, the net magnetization is given by $\overrightarrow{m}= \Delta(\hat{z}\times \overrightarrow{l})$, where Δ is the DMI-set canting angle. This yields the expression $\overrightarrow{\nabla }\cdot {\overrightarrow{m}}_{xy}=\Delta [\hat{z}\cdot (\overrightarrow{\nabla }\times \overrightarrow{l}\,)]$ (Supplementary Section 5). The striking consequence is that the *B*_*z*_ images also offer a projected measure of staggered vorticity, that is, the curl of the Néel vector $\overrightarrow{{{{\mathcal{V}}}}}=\overrightarrow{\nabla }\times \overrightarrow{l}$.

## Characteristic field signatures and vorticity read-out

**Fig. 2:**
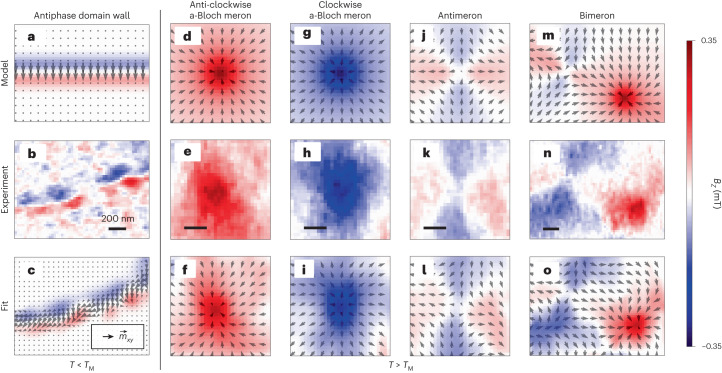
Classification of topological AFM textures via DQM. **a**–**c**, Topological AFM textures observed below *T*_M_. A distinct *B*_*z*_ signature of an ADW calculated (**a**) and measured (**b**) above the sample surface. The reconstructed ${\overrightarrow{m}}_{xy}$ (black arrows) from **b** and its *B*_*z*_ distribution (**c**). **d**–**o**, Topological AFM textures observed above *T*_M_. Calculated (**d**) and measured (**e**) *B*_*z*_-field signatures of an anti-clockwise a-Bloch meron and the reconstructed ${\overrightarrow{m}}_{xy}$ (black arrows) of **e** and its associated *B*_*z*_ (**f**). Similarly, the calculated and measured *B*_*z*_ signatures and the ${\overrightarrow{m}}_{xy}$ reconstruction of a clockwise a-Bloch meron, an antimeron and a bimeron are given in **g**–**i**, **j**–**l** and **m**–**o**, respectively. Supplementary Section 7 provides the details and limitations of ${\overrightarrow{m}}_{xy}$ reconstruction. Scale bars, 200 nm.

After establishing the relationship between *B*_*z*_ and ${\overrightarrow{m}}_{xy}$, next we show that the images obtained in Fig. 1 are produced by AFM antiphase domain walls (ADWs), merons, antimerons and bimerons—consistent with recent observations in α-Fe_2_O_3_ (ref. ^18^). Below *T*_M_, we model the *B*_*z*_ images with a linear AFM domain wall^18^, characterized by width *w* and phase *ξ*_a_ (Supplementary Section 5.1). The phase *ξ*_a_ controls the spatial variation of $\overrightarrow{l}$, resulting in an AFM Néel (a-Néel) or an AFM Bloch (a-Bloch) ADW profile for (*ξ*_a_ = 0, π) and (${\xi }_{{\rm{a}}}=\frac{\uppi }{2}$, $\frac{3\pi }{2}$), respectively. For a linear ADW profile centred at *x* = 0 along the *x* axis, $\overrightarrow{\nabla }\cdot {\overrightarrow{m}}_{xy}=$ ${m}_{\Delta }\left(\frac{\uppi }{w}\right)\sin\left(\frac{\uppi x}{w}\right)\sin ({\xi }_{\rm{a}})$ for $| x| \le \frac{w}{2}$, and zero elsewhere^18^ (Supplementary Section 5.1). Hence, we expect ADWs to display a sinusoidal profile in $\overrightarrow{\nabla }\cdot {\overrightarrow{m}}_{xy}$ and in *B*_*z*_, with zero crossing at the centre, and amplitude and sign modulated by sin(*ξ*_a_). In particular, an a-Néel ADW will not yield a *B*_*z*_ signal as $\overrightarrow{\nabla }\cdot {\overrightarrow{m}}_{xy}=0$, whereas an a-Bloch counterpart will show the maximal signal. Based on equation (1), these characteristics are reflected in the calculated *B*_*z*_ image of an ADW model in Fig. 2a (Supplementary Section 5.4), assuming a phase of *ξ*_a_ = π. The measured *B*_*z*_ image (Fig. 1i) and a close-up image (Fig. 2b) capture the signature zero crossing of an ADW. DQM also reveals variations in *B*_*z*_ along the wall boundary, capturing the spatially varying phase *ξ*_a_ in α-Fe_2_O_3_ (ref. ^18^). This phase dependence of *B*_*z*_ allows the unambiguous identification of the zero-signal sections along the wall with varying *ξ*_a_ as a-Néel ADWs. Figure 2c presents a reconstruction of the ${\overrightarrow{m}}_{xy}$ distribution of the multi-chiral ADW (illustrated by arrows), obtained by fitting *B*_*z*_ to the data in Fig. 2b through systematic regularization (Supplementary Section 7).

In contrast, DQM at 300 K captures larger spatial features of a strong *B*_*z*_ signal (Fig. 1j). In the above-*T*_M_ regime, we anticipate finite net magnetization forming whirling topological structures, such as multi-chiral merons and antimerons^18^, as well as topologically trivial in-plane domain walls. Topological textures can be characterized by the topological charge ${{{\mathcal{Q}}}}$ and topological winding ${{{\mathcal{N}}}}$. Each AFM texture produces a distinctive *B*_*z*_ signal, allowing us to develop a systematic procedure to differentiate them (Supplementary Section 5). Here we focus on 2D topological textures and model isolated (anti)merons based on a linear ansatz^18,33^, described by phase *ξ*_a_ and winding number ${{{\mathcal{N}}}}$ (Supplementary Section 5.3). The corresponding divergence in polar coordinates (*r*, *ϕ*) is $\overrightarrow{\nabla }\cdot {\overrightarrow{m}}_{xy}={m}_{\Delta }\sin \left(\phi (1-{{{\mathcal{N}}}})-{\xi }_{a}\right)f(r)$, where *f*(*r*) is a radial function dependent on the (anti)meron phase (Supplementary Section 5.3). A meron (${{{\mathcal{N}}}}=+1$) produces a radially symmetric *B*_*z*_ distribution about its core with magnitude and polarity controlled by sin(*ξ*_a_). Analogous to ADWs, a-Néel merons (*ξ*_a_ = 0, π) are divergence-free and exhibit *B*_*z*_ = 0, whereas a-Bloch counterparts $\Big({\xi }_{\rm{a}}=\frac{\uppi }{2}$, $\frac{3\uppi }{2}\Big)$ show the maximal *B*_*z*_ amplitude. In contrast, for an antimeron (${{{\mathcal{N}}}}=-1$), the *B*_*z*_ distribution is two-fold symmetric and *ξ*_a_ controls an azimuthal offset. The calculated *B*_*z*_ images in Fig. 2d,g,j of the a-Bloch meron model of both polarities and the antimeron model reinforce these observations (Supplementary Section 5.4). Thus, DQM unambiguously reveals the topological winding number ${{{\mathcal{N}}}}$ and staggered vorticity $\overrightarrow{{{{\mathcal{V}}}}}$ for each spin texture. DQM cannot distinguish the sign of the topological charge of the spin texture due to the vanishing canted moment at its core. Figure 2e,h,k presents the measured *B*_*z*_ images of an anti-clockwise a-Bloch meron (${{{\mathcal{N}}}}=+1,{\xi }_{\rm{a}}=\uppi /2$), a clockwise a-Bloch meron (${{{\mathcal{N}}}}=+1,{\xi }_{\rm{a}}=3\uppi /2$) and an antimeron (${{{\mathcal{N}}}}=-1$), respectively, in good agreement with their modelled counterparts. Further, in Fig. 2f,i,l, we reconstruct the ${\overrightarrow{m}}_{xy}$ distributions and use them to calculate the measured *B*_*z*_ image for each texture discussed above (Supplementary Section 7). Given the density of spin textures evident in Fig. 1j, the reconstructed ${\overrightarrow{m}}_{xy}$ approach better captures the finer details of the measured *B*_*z*_ images in the absence of true isolation. We note that although several simplifying steps are considered in our magnetization reconstruction (Supplementary Section 7), the insights presented in the main text, including those on staggered vorticity, remain valid. We further note that the density of (anti)merons can be reduced via meron–antimeron annihilation mediated by an external in-plane magnetic field^18^ (Supplementary Section 8). Finally, a meron and an antimeron in close proximity can form a stable bimeron. Figure 2m displays the corresponding calculated *B*_*z*_ image of an isolated bimeron model, whereas Fig. 2n shows the measured *B*_*z*_ image of one such occurrence. Similarly, Fig. 2o displays the calculated *B*_*z*_ image from its reconstructed ${\overrightarrow{m}}_{xy}$. Although (anti)merons are always topologically protected, this is not necessarily true for meron–antimeron pairs. Labelling a meron–antimeron pair as topologically protected would require the knowledge of the topological charge sign of its constituents^29,34^.

## Emergent magnetic charge

The fact that DQM provides a direct measure of $\overrightarrow{\nabla }\cdot {\overrightarrow{m}}_{xy}$ creates a unique opportunity to consider a magnetic analogue of the electric Gauss’s law. Namely, the non-zero divergence of magnetization manifests the existence of an areal magnetic charge density via ${\sigma }_{{\rm{m}}}=-t(\overrightarrow{\nabla }\cdot {\overrightarrow{m}}_{xy})$. Here ${\overrightarrow{m}}_{xy}$ is independent of *t*—valid in the thin-film limit^18^. Therefore, AFM textures in α-Fe_2_O_3_ have associated emergent magnetic charge distributions, which locally act as sources or sinks of the magnetic field. We can define a formal duality relation that connects the magnetic charge density *σ*_m_ to the staggered vorticity $\overrightarrow{{{{\mathcal{V}}}}}$ via

2 $${\sigma }_{m}/t=-\overrightarrow{\nabla }\cdot {\overrightarrow{m}}_{xy}=\Delta (\hat{z}\cdot \overrightarrow{{{{\mathcal{V}}}}}),$$

which scales with sin(*ξ*_a_), highlighting the influence of the texture phase. Crucially, retrieving the emergent charge density *σ*_m_ only requires the Fourier deconvolution of the measured *B*_*z*_ images from the transfer function *α*_*xy*_ (Supplementary Section 4.2). We can also perform a downward (upward) continuation^35^ (Supplementary Section 4.2) of the planar $\overrightarrow{B}$ distribution captured in Fig. 2b,e,h,k,n, down to (away from) the sample surface. This allows a three-dimensional visualization of $\overrightarrow{B}$ ($=\overrightarrow{H}$ in a vacuum) in the volume above the magnetic charge distribution. Note that this charge and field retrieval process is independent of the ${\overrightarrow{m}}_{xy}$ reconstruction and the linear meron model assumption.

**Fig. 3:**
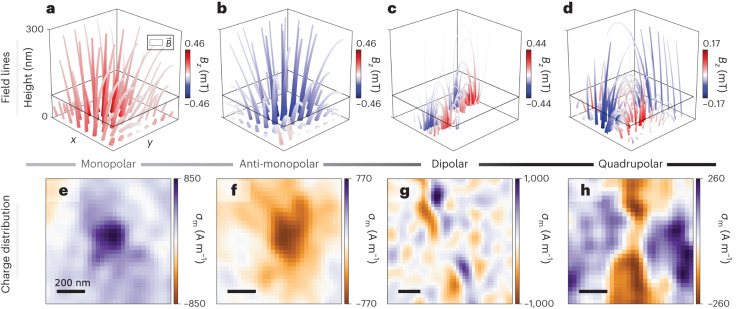
Emergent magnetic charge distributions. **a**–**d**, Three-dimensional visualization of $\overrightarrow{B}$ in the volume above an assortment of topological AFM textures. The streamtubes illustrate the magnetic field lines of $\overrightarrow{B}$ above an anti-clockwise a-Bloch meron (**a**), a clockwise a-Bloch meron (**b**), an ADW (**c**) and an antimeron (**d**). The girth and colour of each streamtube vary with the magnetic field norm $|\vec{B}|$ and the *z* component of field (*B*_*z*_), respectively. **e**–**h**, Magnetic charge density (*σ*_m_) distributions retrieved from the downward continuation of **a**–**d** reveal a magnetic monopolar (**e**), anti-monopolar (**f**), dipolar (**g**) and quadrupolar (**h**) charge character associated to an anti-clockwise a-Bloch meron, clockwise a-Bloch meron, ADW and antimeron, respectively. Scale bars, 200 nm.

Figure 3a–d illustrates the field lines of $\overrightarrow{B}$ above the (anti-)clockwise a-Bloch meron, ADW and antimeron. For the two Bloch merons, $\overrightarrow{B}$ is consistent with the profile expected for spatially extended sources and sinks of the magnetic field emanating from a monopolar distribution, which we retrieved via equation (2) in Fig. 3e,f. This implies that a-Bloch merons host a class of emergent monopoles piggybacking on the topologically protected AFM textures. Interestingly, such monopolar magnetic distributions are not observed in ferromagnetic materials, as the presence of long-range demagnetizing fields favours divergence-free Bloch textures. In our case, we are freed from this constraint due the presence of a weak demagnetizing contribution relative to the stronger AFM exchange in α-Fe_2_O_3_. In contrast to merons, the ADW in Fig. 3c and the antimeron in Fig. 3d are associated with *σ*_m_ distributions that exhibit dipolar and quadrupolar characters, respectively (Fig. 3g,h). Finally, we emphasize that the observation of emergent monopoles is fully consistent with the modelling of AFM topological textures in Fig. 2 and does not violate Maxwell’s equation as they are, in fact, sinks and sources of the $\overrightarrow{H}\,$ field. Although $\overrightarrow{\nabla }\cdot \overrightarrow{H}\ne 0$ in the material, the condition of $\overrightarrow{\nabla }\cdot \overrightarrow{B}=0$ is still conserved since $\overrightarrow{B}={\mu }_{0}(\overrightarrow{H}+\overrightarrow{m})$ and in the absence of any free electric current, $\nabla \cdot \overrightarrow{H}=-\nabla \cdot \overrightarrow{m}$. Therefore, when probing the fields outside the material, where ${\mu }_{0}\overrightarrow{H}=\overrightarrow{B}$, one sees a field $\overrightarrow{B}$ that appears as if it is emerging from sources and sinks given by $\overrightarrow{\nabla }\cdot \overrightarrow{m}$ (refs. ^26,36,37^).

**Fig. 4:**
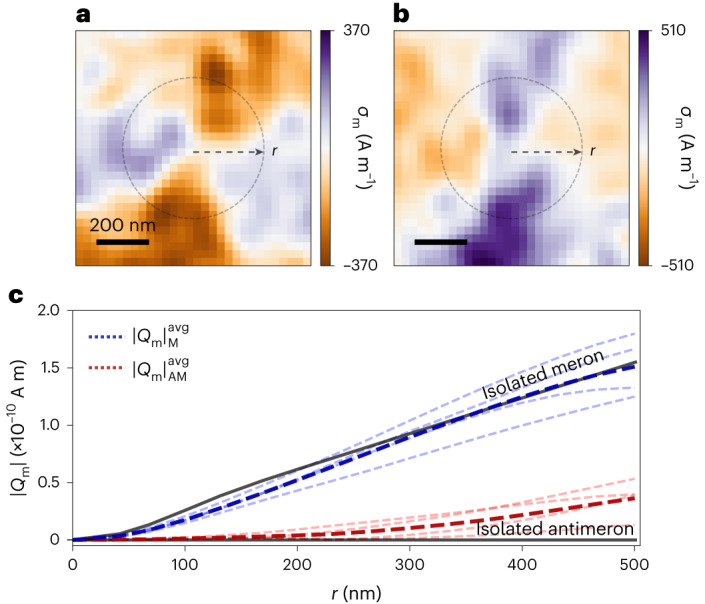
Scaling of 2D integrated magnetic charges. **a**,**b**, Reconstructed magnetic charge distribution (*σ*_m_) of two experimentally observed antimerons (AM 1 and AM 2) with slightly distorted quadrupolar characters. AM 1 and AM 2 display a bias towards positive and negative charges, respectively. The dashed circle in **a** and **b** illustrates the circular integration area *S* of radius *r*, centred at the core of the antimeron, to obtain |*Q*_m_|. Scale bars, 200 nm. **c**, Experimentally retrieved magnitude of the total integrated magnetic charge of multiple merons (|*Q*_m_|_M_, light-blue dashed curves) and antimerons (|*Q*_m_|_AM_, light-red dashed curves) plotted as a function of integration radius *r*. Their average experimental |*Q*_m_| profiles, namely, $| {Q}_{\rm{m}}{| }_{{{{\rm{M}}}}}^{{{{\rm{avg}}}}}$ and $| {Q}_{\rm{m}}{| }_{{{{\rm{AM}}}}}^{{{{\rm{avg}}}}}$, are represented by dark-blue and dark-red curves, respectively. The solid black curves plot the theoretically predicted *Q*_m_ radial dependence based on equation (3) for isolated merons and antimerons.

Based on the above analysis, it is tempting to attribute a non-zero net monopolar charge to isolated a-Bloch merons, quantified by *Q*_m_ ≡ ∫_*S*_*σ*_m_d*S* within area *S*. We pick a circular integration area *S* of radius *r* centred on a given spin texture. Figure 4a,b illustrates an example in the case of antimerons. The (1/*r*) dependence of $\overrightarrow{\nabla }\cdot {\overrightarrow{m}}_{xy}$ for 2D magnetic charges hosted by spin textures then yields (Supplementary Section 5.9)

3 $${Q}_{\rm{m}}(r)=\left\{\begin{array}{ll}2\uppi \,{{m}}_{\Delta }\,\sin ({\xi }_{\rm{a}})\,\sin \left(\frac{\uppi r}{2{R}_{\rm{M}}}\right)\,r\,t\quad &,{{{\mathcal{N}}}}=+1,\,r\le {R}_{\rm{M}}\\ 2\uppi \,{m}_{\Delta }\,\sin ({\xi }_{\rm{a}})\,r\,t\quad &,{{{\mathcal{N}}}}=+1,\,r > {R}_{\rm{M}}.\\ 0\quad &,{{{\mathcal{N}}}}=-1\end{array}\right.$$

Figure 4c presents the radial dependence of *Q*_m_ for four measured merons (light-blue dashed curves) and antimerons (light-red dashed curves), whereas the dark-blue (red) dashed curve is the average *Q*_m_ radial dependence for merons (antimerons). For an isolated linear meron model, *Q*_m_ scales linearly with *r*, and the measured *Q*_m_ radial dependence is in agreement with this. *Q*_m_ itself is not a topological invariant, as a smooth transformation of an a-Bloch meron to an a-Néel meron would tune *Q*_m_ from non-zero to zero. For an isolated antimeron, the two-fold rotational symmetry ensures *Q*_m_ = 0 for all *r* values (Supplementary Section 5.9) and at short distances, the experimental *Q*_m_ value of an antimeron agrees well with this prediction. Beyond measurement-induced variations, deviation from the strict *Q*_m_ = 0 condition arises when an antimeron is influenced by neighbouring spin textures. This reduces the two-fold symmetry and reveals a finite *Q*_m_. Figure 4a,b captures this reduced symmetry as well as the consequential bias towards negative and positive *Q*_m_, respectively. Finally, as *r* goes to infinity, the integration area would overlap with the surrounding spin textures, leading to a further deviation from the *Q*_m_ = 0 condition.

The assumption that we have a collection of isolated spin textures oversimplifies the reality. Although the AFM topological textures are mesoscopically discernible and thus appear localized, they are, in fact, the constituents of the complex multi-textural ensemble that interacts via a 2D magnetic charge canvas. The magnetic charge per constituent is not just dictated by their nature as merons and antimerons, but modified through their interaction with other constituents. For example, an isolated meron and antimeron pair forming an AFM bimeron (Fig. 2n) would have a non-zero *Q*_m_, whose sign is predominantly determined by its meron (Supplementary Section 5.9). However, this clearly cannot be the case for a bimeron embedded in a uniform magnetization field in the far field (Supplementary Section 5.10), since the divergence theorem ensures that *Q*_m_ = 0. This indicates that the interaction among AFM textures produces additional magnetic charge density away from the cores and highlights the interactive nature of this multi-textural ensemble.

## Discussion and outlook

Our ability to identify the duality between topological AFM textures and magnetic charges is due to the direct read-out of staggered vorticity enabled by DQM. Specifically, the NV centre senses the amplitude of the magnetic field projected onto the NV axis, allowing us to deduce the three field components via their linear dependence. This enables us to independently reconstruct the magnetic charge and local magnetization. As such, we go beyond detecting antimerons and merons, to further distinguish between clockwise and anti-clockwise a-Bloch components, which otherwise appear indistinguishable in X-ray dichroic images. Our imaging approach can be extended to a wider family of topological textures, including skyrmions, a-Néel merons and bimerons, as well as distorted AFM textures that are otherwise divergence-free (Supplementary Section 5.6–5.8), relevant for topological AFM circuitry^7,27,38^.

Although haematite provides favourable conditions for DQM imaging due to spin canting, it is, by no means, unique among AFMs in possessing a weak net magnetization or quasi-isotropic spins in two dimensions. It may be possible to observe topological phenomenology in similar canted AFMs, including orthoferrites, orthochromites and iron borate^39–42^. In compensated AFMs without bulk DMI, staggered spin textures can also generate a local net magnetization, either statically or dynamically^8,43,44^. Moreover, DQM can be useful in detecting preferential vorticity in ultrathin films induced by interfacial interactions—a key requirement for applications in topological spintronics^27,28^.

The reported duality between magnetic charges and topological AFM textures sheds light on a new class of materials hosting 2D monopolar physics in contrast with other systems that harbour emergent magnetic monopoles, such as the pyrochlore spin ice^26^. Although intriguing, monopoles in spin ice are intrinsically distinct, as they have an underlying gauge charge, which is topological and quantized. Conversely, the emergent magnetic charges in haematite are 2D, not quantized and are topological in the sense that they dress topological AFM textures underpinning them. We have demonstrated that haematite supports a rich tapestry of interacting magnetic charge distributions that could open up new and complementary ways to detect, manipulate and functionalize—via their magnetic charge—AFM topological textures. Our capability to classify different AFM spin textures could be combined with conventional spin manipulation techniques such as spin torques, allowing for read-out and write-in schemes. Realizing this in a complex manifold of states endowed with highly nonlinear interactions could be attractive for unconventional computing^1,2^. Finally, the intriguing physical insights revealed in α-Fe_2_O_3_ are a testament to the relevance and potential of DQM as a versatile table-top platform to explore emergent phenomena in AFMs and other quantum materials.

## Online content

Any methods, additional references, Nature Portfolio reporting summaries, source data, extended data, supplementary information, acknowledgements, peer review information; details of author contributions and competing interests; and statements of data and code availability are available at 10.1038/s41563-023-01737-4.

### Supplementary information

Supplementary Information Supplementary Sections 1–10, Figs. 1–35, Equations (1)–(39) and discussion.

## Data Availability

All data needed to evaluate the conclusions in the paper are available in this Article or its Supplementary Information. The data that support the findings of this study are available from the corresponding authors upon reasonable request.
