# Supplementary material for: Revealing emergent magnetic charge in an antiferromagnet with diamond quantum magnetometry
Source: Nat Mater. 2023 Dec 5;23(2):205–11. doi: 10.1038/s41563-023-01737-4 (PMC10837077; doi:10.1038/s41563-023-01737-4)
Supplement: Supplementary file 1 — Supplementary Sections 1–10, Figs. 1–35, Equations (1)–(39) and discussion. [file 41563_2023_1737_MOESM1_ESM.pdf]

nature materials

Article

https://doi.org/10.1038/s41563-023-01737-4

Revealing emergent magnetic charge in an antiferromagnet with diamond quantum magnetometry

In the format provided by the authors and unedited

Contents

S1 Material Growth and Characterisation 2

S2 Setup and Imaging Details 2

S3 Sensor Characterisation 3
S3.1 NV-axis Orientation 3
S3.2 NV-to-Sample Distance 4

S4 Magnetic Field Analysis 5
S4.1 Magnetic Field Components Above Sample Surface 5
S4.2 Downward (Upward) Continuation and Charge Retrieval 6

S5 Magnetic Models 7
S5.1 Magnetic Field and Charge of AFM Antiphase Domain Walls 8
S5.2 Magnetic Field and Charge of AFM In-plane Domain Walls 8
S5.3 Magnetic Field and Charge of AFM Merons and Antimerons 9
S5.4 Magnetic Field Models of Various AFM Entities 10
S5.5 Distinguishing Topological and non-topological AFM textures 12
S5.6 Invisibility of Divergence-free Textures 14
S5.7 Magnetic Field and Charge of AFM Bimerons 15
S5.8 Magnetic Field and Charge of AFM Skyrmions 18
S5.9 Integrated Magnetic Charge of AFM Merons and Antimerons 20
S5.10 Charge Variability of AFM Bimerons 21
S5.11 Topological Numbers 23

S6 Meron Core Size Analysis 24

S7 Retrieving $\vec{m}_{xy}$ 25
S7.1 Simplification of Retrieval Problem 26
S7.2 Regularisation 26
S7.3 Fitting of Antiphase Domain Walls 27
S7.4 Fitting of (Anti)Merons and Bimerons 29
S7.5 Fitting Multiple Merons and Antimerons 34
S7.6 Field Imaging and Magnetisation Reconstruction of α-Fe_2O_3 37
S7.7 Additional Experimental Reconstruction Data 41

S8 Reversal Field Imaging 42

S9 Additional Data 44

S10 Dipolar Tensor 45

S1 Material Growth and Characterisation

Epitaxial α-Fe_2O_3 films were pulsed-laser deposited on (0001)-oriented single crystalline α-Al_2O_3 substrates (CrysTec GmbH), using a KrF excimer laser at 248 nm. X-ray reflectometry indicated a film thickness $t = 25$ nm. A detailed fabrication recipe can be found in our previous studies (*1,2*). No capping overlayer was used in this study. Bulk magnetometery was performed using a Superconducting Quantum Interference Device magnetometer (Quantum Design). The temperature dependence of the canted moment, shown in Fig. S1, reveals a Morin transition in the range of $200 - 220$ K such that the magneto-crystalline anisotropy changes from in-plane ($T > T_M$) to out-of-plane ($T < T_M$).

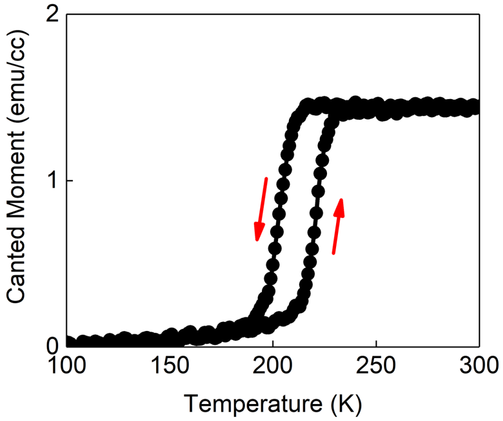

Figure S1: Magnetometry characterisation. Temperature dependence characterisation (M-T) with an in-situ 50 mT in-plane field. Arrows indicate the direction of the temperature sweep.

S2 Setup and Imaging Details

The diamond quantum magnetometry (DQM) setup consists of an electrical readout atomic force microscope in a closed-cycle cryostat (attoDRY1000, Attocube systems) integrated with a home-built confocal setup. For magnetic field sensing, CW-ODMR was employed for sen-

sor characterisation, and switched to pulsed-ODMR for the imaging of α-Fe_2O_3 to improve sensitivity. The diamond sensor used in our measurements (Qzabre AG) contains Nitrogen-15 ($I = 1/2$) thereby giving two hyperfine resonances per electron spin resonance with a 3 MHz splitting that is easily detectable with pulsed-ODMR. The on-axis field magnitude is determined from the ODMR frequency splitting via the weak field approximation (*3*), where the influence of the off-axis field on the zero-field splitting is neglected. Microwave excitation is delivered via a copper wire pulled across the sample surface. Imaging is conducted with the diamond sensor positioned $\sim 50\ \mu\mathrm{m}$ away from the wire antenna. A Tektronix AWG (70002A) was programmed to provide the pulse sequences necessary for pulsed-ODMR. Typical scans have a pixel size of ~ 33.3 nm, with an averaging time per pixel of $50 - 100$ ms at both 4 K and 300 K. The total time for a $5 \times 5\ \mu\mathrm{m}^2$ image is approximately twelve hours, with the retrace speed being ≤ 0.1 Hz to avoid physical damage to the sample surface by the diamond pillar. The fast-scan direction coincides with the x-axis of our images.

S3 Sensor Characterisation

S3.1 NV-axis Orientation

NV-axis orientation with reference to the lab frame is characterised by applying an external magnetic field $\vec{B}(B_0, \theta_B, \phi_B)$ using a three-axis Helmholtz coil. We characterise the NV orientation by obtaining ODMR spectra while holding the B-field at a constant magnitude B_0 and polar θ_B (azimuthal ϕ_B) angle and sweeping ϕ_B (θ_B) (details are found in (*4*)). The NV-axis orientation characterisation of the diamond sensor used for imaging in Figs 1-3 in the main text (fig.S2), shows $\theta_{NV} = 120 \pm 4^{\circ}$ and a $\phi_{NV} = 96 \pm 4^{\circ}$.

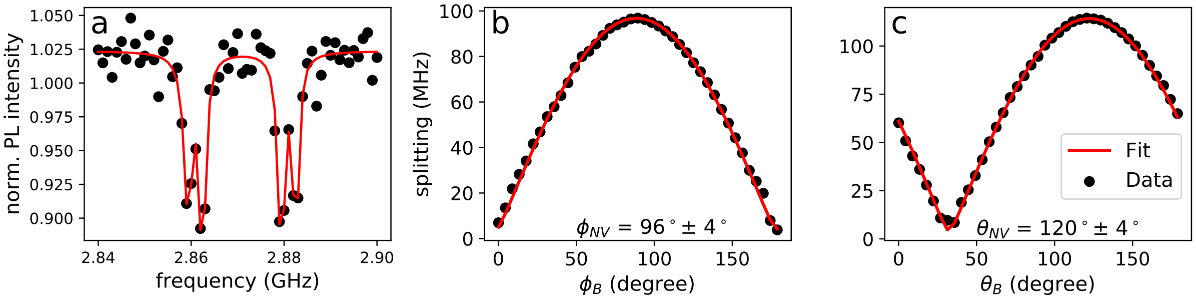

Figure S2: NV orientation characterisation. (a) ODMR with 0.5 mT applied along the NV axis showing the Zeeman-split resonances with additional hyperfine splitting due to the host ^{15}N. ODMR spectrum as a function of (b) ϕ_B with θ_B fixed at 180° and (c) θ_B with $\phi_B = 96°$. The maximum splitting in (b) determines $\phi_{NV} = 96°$ which is then used in (c) to identify $\theta_{NV} = 120°$.

S3.2 NV-to-Sample Distance

The NV-to-sample distance d_{NV} is determined by measuring the magnetic field emitted across the edge of an out-of-plane magnetic material with the sensor of interest. Our calibration sample is a CoFeB magnetic strip (details in (*4*)) with a magnetisation that remains saturated at remanence. This allows for accurate d_{NV} value extraction following the proposal in (*5*). An example ODMR linescan across the edge of the magnetic strip, obtained with the diamond sensor used for imaging in Figs. 1-3 in the main text, is given in Fig. S3 (a). A distribution of extracted d_{NV} values from multiple ODMR linescans, such as the one in (a), is given in Figure S3 (b), resulting in an average d_{NV} value of 70 ± 6 nm which sets the effective spatial resolution of our imaging. This is sufficient to study the AFM topological textures and piggybacked charges in the study, whose length scales are larger.

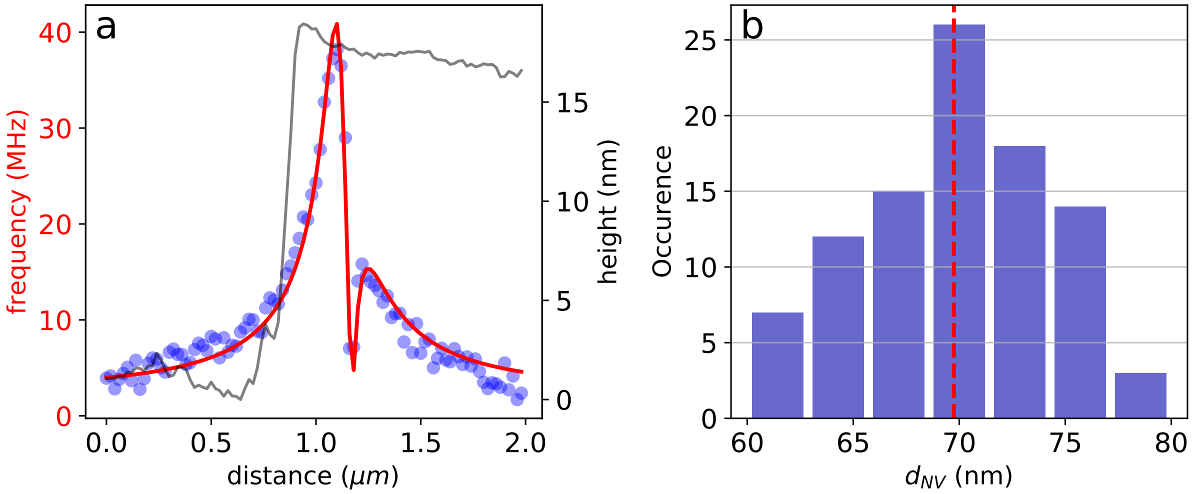

Figure S3: NV-to-sample distance characterisation. (a) ODMR spectrum across the edge of a CoFeB magnetic strip plotted on top of the corresponding topography. From the fit one can extract the NV-to-sample distance. (b) Height distribution extracted from 100 line scans across the edge, giving an average NV-to-sample distance of 70 ± 6 nm.

S4 Magnetic Field Analysis

S4.1 Magnetic Field Components Above Sample Surface

Assuming a density of magnetic dipoles $\vec{m}(x,y)$ of thickness *t*, the magnetic field $\vec{B}(x,y)$ generated at a distance *d* above the sample surface (source-free region) can be described in Fourier space via the dipolar tensor (*6–8*):

$$\begin{pmatrix} \tilde{B}_x(k_x,k_y,d) \\ \tilde{B}_y(k_x,k_y,d) \\ \tilde{B}_z(k_x,k_y,d) \end{pmatrix} = -\frac{\mu_0}{2} e^{-kd}(1-e^{-kt}) \begin{pmatrix} k_x^2/k^2 & k_xk_y/k^2 & ik_x/k \\ k_xk_y/k^2 & k_y^2/k^2 & ik_y/k \\ ik_x/k & ik_y/k & -1 \end{pmatrix} \begin{pmatrix} \tilde{m}_x(k_x,k_y) \\ \tilde{m}_y(k_x,k_y) \\ \tilde{m}_z(k_x,k_y) \end{pmatrix}. \quad \text{(S1)}$$

In the absence of a canted m_z component in α-Fe_2O_3, we have:

$$\tilde{B}_z(k_x, k_y, d) = -\frac{\mu_0}{2}e^{-kd}(1 - e^{-kt})\left(\frac{ik_x\tilde{m}_x + ik_y\tilde{m}_y}{k}\right), \tag{S2}$$

$$B_z = \mathscr{F}^{-1}\left[\left(-\frac{\mu_0 e^{-kd}(1 - e^{-kt})}{2k}\right)(ik_x\tilde{m}_x + ik_y\tilde{m}_y)\right] = \alpha_{xy}(d, t) * \vec{\nabla} \cdot \vec{m}_{xy}. \tag{S3}$$

In equation S3 we use the convolution theorem, and define $\alpha_{xy}(d,t) \equiv \mathscr{F}^{-1}(-\frac{\mu_0 e^{-kd}(1-e^{-kt})}{2k})$ and $\mathscr{F}$ and $\mathscr{F}^{-1}$ are the Fourier transform and its inverse. In real space, one can intuitively interpret equation S3 as the convolution of a point spread function, α_{xy} (*9*), with the divergence of the in-plane canted magnetisation, $\vec{\nabla}\cdot\vec{m}_{xy}$. In fact, as the distance d above the sample surface increases, spatial features of $\vec{\nabla} \cdot \vec{m}_{xy}$ get increasingly 'blurred out', decreasing the effective spatial resolution of the NV sensor. In this work, features in $\vec{\nabla} \cdot \vec{m}_{xy}$ that are smaller than d_{NV} (70 nm in our study) will not be resolved. For an a-Bloch meron, this would mean that we are only able to reconstruct $\vec{m}_{xy}$ accurately down to 70 nm. A general discussion involving all components of the magnetisation can be found in (*9*).

S4.2 Downward (Upward) Continuation and Charge Retrieval

Given the constrains set out in section S4.1 and following equation S1, one can write the relation (*10, 11*):

$$\tilde{\vec{B}}(d + \Delta d) = e^{-k\Delta d}\tilde{\vec{B}}(d), \tag{S4}$$

where $\tilde{\vec{B}} = \mathscr{F}(\vec{B})$. A positive or negative Δd in equation S4 corresponds to an *upward* or *downward* continuation of the B-field distribution captured at d. Notice that when Δd is positive, the exponential term $e^{-k\Delta d}$ acts to smooth out high frequency components and physically translates to losing spatial resolution. In the negative case, the exponential term tends to amplify

high frequency noise present in the measurements. For this reason, we apply a Hanning window with a cut-off frequency at $2\pi/d_{NV}$, which is a common method to suppress the undesired amplification of high-frequency noise (*10, 12, 13*).

By applying the *downward continuation* method down to the sample surface, it is therefore possible to retrieve the $\sigma_m = -t\,\vec{\nabla}\cdot\vec{m}_{xy}$ given by:

$$\sigma_m = -t\,\mathscr{F}^{-1}\big(\tilde{B}_z/\tilde{\alpha}_{xy}(t, d = d_{\mathrm{NV}})\big), \tag{S5}$$

where $\tilde{\alpha}_{xy} = \mathscr{F}(\alpha_{xy})$. This is a deconvolution process with the function α_{xy}.

S5 Magnetic Models

The presence of $\vec{\mathcal{D}}$ along the c-axis in α-Fe_2O_3 (bulk DMI) produces a small in-plane canting of angle Δ in the basal ab-plane, generating a net magnetisation which can be obtained from the local Néel vector using $\vec{m} = \vec{\mathcal{D}} \times \vec{l} = \Delta\hat{\eta} \times \vec{l}$ in the small angle approximation. In α-Fe_2O_3, the unit vector $\hat{\eta} = (0, 0, +1)$ (*14*) is parallel to the c-axis. Since in our case the c-axis is along the z-axis of the lab frame, the expression is simplified to $\vec{m} = \Delta\hat{z} \times \vec{l}$. Note that this condition is strictly enforced in the trigonal symmetry of the paramagnetic phase but is relaxed at lower temperatures due to magneto-elastic interactions, resulting in a small out-of-plane moment, which has hence been ignored here (*15*). With this, we will now describe the spatial distributions of the canted moment in various AFM quasiparticles considering their respective linear ansatz.

Herein, we assume a canted moment $m_\Delta \sim 2 \times 10^3$ A/m, and a canting angle $\Delta \sim 1.1$ mrad. This is consistent with values reported for bulk α-Fe_2O_3 (Table 4.2 of (*16*)) and comparable to the magnetic response of our samples, as discussed in our previous studies

(*1, 2*). In the small angle approximation, the average magnitude of the sublattice magnetisation $M_s = m_\Delta/(2\Delta) \sim 9.1 \times 10^6$ A/m.

S5.1 Magnetic Field and Charge of AFM Antiphase Domain Walls

Assuming a linear ansatz for the antiphase domain wall profile centered at $x = 0$ along the x-axis, with phase ξ_a and width w, we have:

$$\vec{l} = 2M_s \left(\cos\theta\cos\xi_a \;,\; \cos\theta\sin\xi_a \;,\; \sin\theta \right) , \tag{S6}$$

$$\vec{m} = 2\Delta M_s \left(-\cos\theta\sin\xi_a \;,\; \cos\theta\cos\xi_a \;,\; 0 \right) , \tag{S7}$$

$$\text{where,} \quad \theta = \begin{cases} -\frac{\pi}{2} & , \; x < -\frac{w}{2} \\ \frac{\pi x}{w} & , \; |x| \leq \frac{w}{2} \\ +\frac{\pi}{2} & , \; x > \frac{w}{2} \end{cases} .$$

Hence,

$$\vec{\nabla} \cdot \vec{m} = \begin{cases} 2\Delta M_s \left(\frac{\pi}{w}\right) \sin\theta\sin\xi_a & , \; |x| \leq \frac{w}{2} \\ 0 & , \; |x| > \frac{w}{2} \end{cases} . \tag{S8}$$

S5.2 Magnetic Field and Charge of AFM In-plane Domain Walls

Uniform IP AFM domains do not generate any stray fields since their magnetisation would be non-divergent (see main text Eqn. 1). However, in-plane domain walls above the Morin transition, which are expected in α-$Fe_2O_3$to span 60° and 120°, can generate stray fields. Their stray field signature depends on the domain wall width w, winding direction l_0, internal phase

of the domain wall – given by the angular span η_0, and phase offset ϕ_0:

$$\vec{l} = 2M_s \; \Big(\; \cos\big(\Psi + \phi_0\big) \; , \; \sin\big(\Psi + \phi_0\big) \; , \; 0 \; \Big) \; , \tag{S9}$$

$$\vec{m} = 2\Delta M_s \; \Big(\; -\sin\big(\Psi + \phi_0\big) \; , \; \cos\big(\Psi + \phi_0\big) \; , \; 0 \; \Big) \; , \tag{S10}$$

$$\text{where,} \quad \Psi = \begin{cases} -\frac{l_0\eta_0}{2} & , \; x < -\frac{w}{2} \\ \frac{l_0\eta_0 x}{w} & , \; |x| \leq \frac{w}{2} \\ +\frac{l_0\eta_0}{2} & , \; x > \frac{w}{2} \end{cases} \; .$$

Hence,

$$\vec{\nabla} \cdot \vec{m} = \begin{cases} -2\Delta M_s \big(\frac{l_0\eta_0}{w}\big) \cos\left(\Psi + \phi_0\right) & , \; |x| \leq \frac{w}{2} \\ 0 & , \; |x| > \frac{w}{2} \end{cases} \; . \tag{S11}$$

IP domain walls generate different stray fields depending on their internal phase as illustrated in Fig. S5.

S5.3 Magnetic Field and Charge of AFM Merons and Antimerons

Assuming a linear ansatz for an (anti)meron centered at the origin, with phase ξ_a, topological winding number $\mathcal{N}$, and radial size R_M, we have:

$$\vec{l} = 2M_s \; \Big(\; \sin\theta \cos\left(\mathcal{N}\phi + \xi_a\right) \; , \; \sin\theta \sin\left(\mathcal{N}\phi + \xi_a\right) \; , \; \cos\theta \; \Big) \; , \tag{S12}$$

$$\vec{m} = 2\Delta M_s \; \Big(\; -\sin\theta \sin\left(\mathcal{N}\phi + \xi_a\right) \; , \; \sin\theta \cos\left(\mathcal{N}\phi + \xi_a\right) \; , \; 0 \; \Big) \; , \tag{S13}$$

$$\text{where,} \quad \theta = \begin{cases} \frac{\pi r}{2R_M} & , r \leq R_M \\ \frac{\pi}{2} & , r > R_M \end{cases} .$$

Hence,

$$\vec{\nabla} \cdot \vec{m} = \begin{cases} 2\Delta M_s \sin\left((1-\mathcal{N})\phi - \xi_a\right)\left(\frac{\mathcal{N}}{r}\sin\theta + \frac{\pi}{2R_M}\cos\theta\right) & , r \leq R_M \\ 2\Delta M_s \sin\left((1-\mathcal{N})\phi - \xi_a\right)\left(\frac{\mathcal{N}}{r}\right) & , r > R_M \end{cases} . \tag{S14}$$

The last term is referred to as a radial function $f(r)$ in the main text.

S5.4 Magnetic Field Models of Various AFM Entities

We have calculated the magnetic fields generated $d = 70$ nm above $\vec{m}$ grids containing antiphase domain walls in Fig. S4, 60° and 120° in-plane domain walls in Fig. S5, and merons and antimerons in Fig. S6.

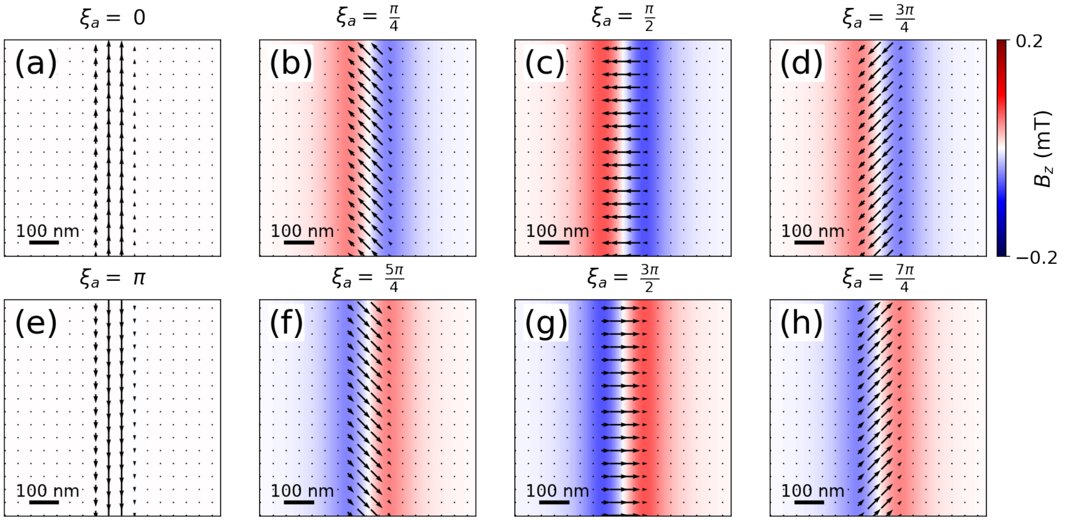

Figure S4: Magnetic field simulation of one-dimensional topological textures. calculated B_z exhibited by ADW models with varying ξ_a. Black arrows illustrate $\vec{m}_{xy}$.

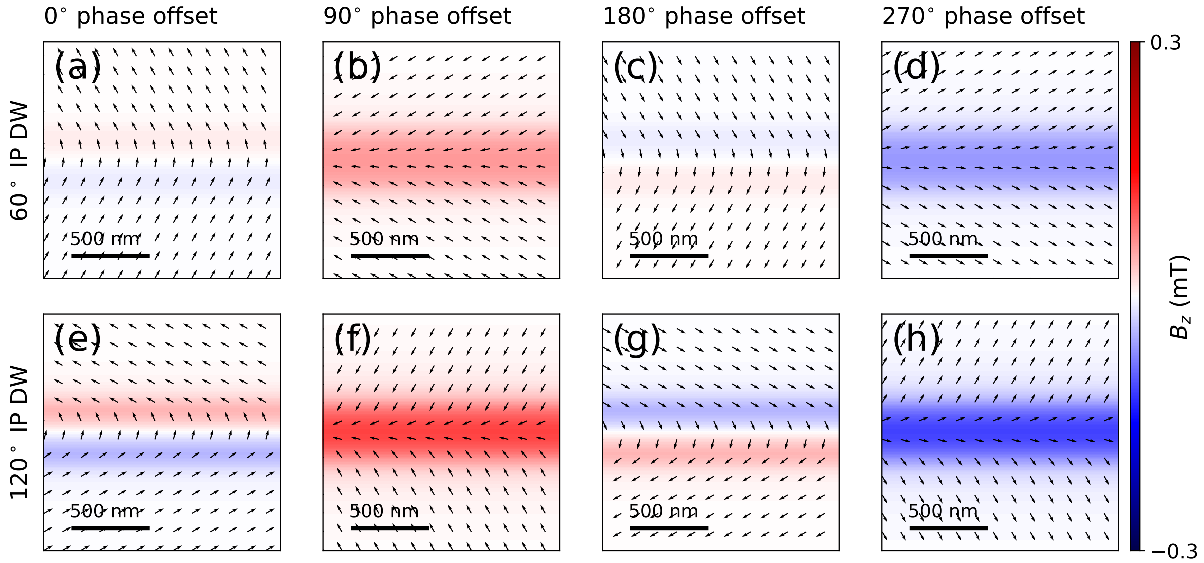

Figure S5: Magnetic field simulation of trivial in-plane domain walls. Calculated B_z exhibited by 60° and 120° in-plane domain wall models with various phase offset. Black arrows illustrate $\vec{m}_{xy}$.

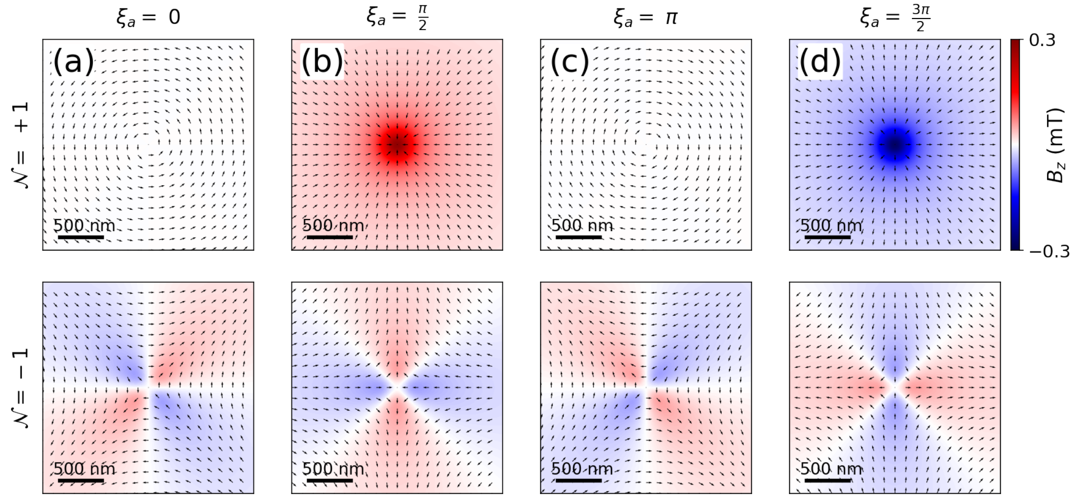

Figure S6: Magnetic field simulation of two-dimensional topological textures. Calculated B_z exhibited by merons ($\mathcal{N} = +1$) and antimerons ($\mathcal{N} = -1$) models with varying ξ_a. Black arrows illustrate $\vec{m}_{xy}$.

S5.5 Distinguishing Topological and non-topological AFM textures

Above the Morin transition, topological textures are always intertwined with IP domain walls – in fact, each topological texture is a ‘pinch-point’ of IP domain walls (*1, 17*). It is therefore crucial to be able distinguish them apart. Whilst IP domain walls can generate stray fields (fig. S5), their strength, distribution, and evolution are markedly different from counterparts generated by merons and antimerons (Fig. S6). In the following we outline the salient differences and how one can distinguish them:

- Locally, topological merons and antimerons are two-dimensional entities which produce confined stray fields, characteristic of monopolar and quadrupolar distributions. Alternatively, IP domain walls are deconfined quasi-one-dimensional trivial textures. This is illustrated in Fig. S7 using the linear meron ansatz.

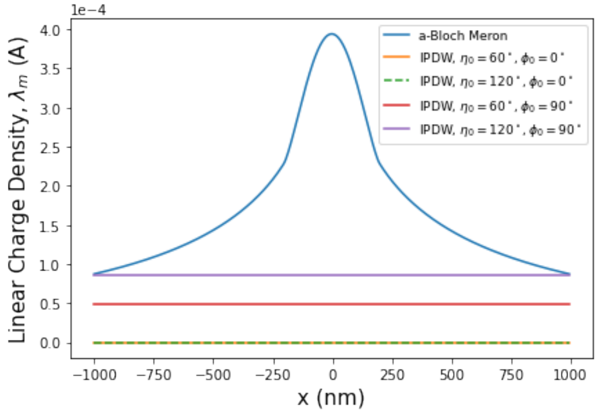

Figure S7: Linear charge profile merons and IP domain walls. The linear charge profile λ_M is the integrated charge along the y-axis given as a function of the x-axis of the meron in Fig. S6 and the IP domain walls (IPDW) in Fig. S5. The λ_M of the meron and IPDWs reveal their respective confined and deconfined nature.

- The maximum stray field magnitude generated by merons and domain walls is (i) inversely proportional to their characteristic length scale (e.g., meron core radius; IP domain wall width, W) and (ii) directly proportional to the total angle they span in spin

space (π for meron; η_0 for IP domain wall angle) and is given by:

$$B_{z,\mathrm{meron}}^{\max} \propto |\vec{\nabla} \cdot \vec{m}_{xy}|_{\mathrm{meron}}^{\max} = 2\Delta M_s \left(\frac{\pi}{R_M}\right) \tag{S15}$$

$$B_{z,\mathrm{IPwall}}^{\max} \propto |\vec{\nabla} \cdot \vec{m}_{xy}|_{\mathrm{IPwall}}^{\max} = 2\Delta M_s \left(\frac{\eta_0}{W}\right) \tag{S16}$$

Since merons trap out-of-plane cores, the anisotropy energy penalty causes these cores to be small (*1*). On the other hand, IP domain walls are much wider as they do not have out-of-plane regions and are only limited by the very weak basal anisotropy in α-Fe_2O_3 (*18*). This causes $R_M < W$, at temperatures well above the Morin transition, which is the case at 300 K. Moreover, since IP domain walls are expected to have $\eta_0 \leq \pi$. Consequently, the magnitude of the stray field generated by a-Bloch merons at their core is significantly more intense, by a factor of ~ 2 compared to trivial textures. This allows unambiguous identification of topological meron cores.

- Although both topological and trivial textures are present above the Morin transition, application of in-plane magnetic fields results in the large-scale annihilation of merons and antimerons only. This triggers the selective elimination of strong and confined monopolar stray fields, leaving behind trivial textures, as illustrated in SI section S8 and our previous work (*1, 17*). This experimentally evinces the different evolution of topological and non-topological entities.

- As for antimerons, the quadrupolar stray field distribution with a zero-field cross pattern (Fig. S6) while distinct from IP domain walls (Fig. S5), can be challenging to disambiguate in isolation. For this reason, we restrict antimeron identification and fitting to regions in the immediate proximity of strong monopolar field distributions generated by merons. This is justified, given that merons and antimerons occur with equal probabil-

ity after executing a Kibble-Zurek transition (*1, 17*) and that, based on our X-ray work, meron-antimeron pairs are extremely common.

S5.6 Invisibility of Divergence-free Textures

For a divergence-free texture (i.e. $ik_x\tilde{m}_x + ik_y\tilde{m}_y = 0$), the z-component of the field in Eqn. S2 vanishes, rendering it invisible to magnetic field detection. This is further illustrated in Figs. S8 and S9, where ADWs and merons turn invisible when $\xi_a = 0, \pi$. In reality, textures are never perfectly isolated, and the distortion produced by the proximity of other textures will make even a-Néel meron visible through the resulting magnetic field. This is illustrated in figure S10, which display the magnetic field of an a-Néel meron undergoing progressive geometrical distortion, and will be further discussed in Fig. S15 in the context of a-Néel bimerons.

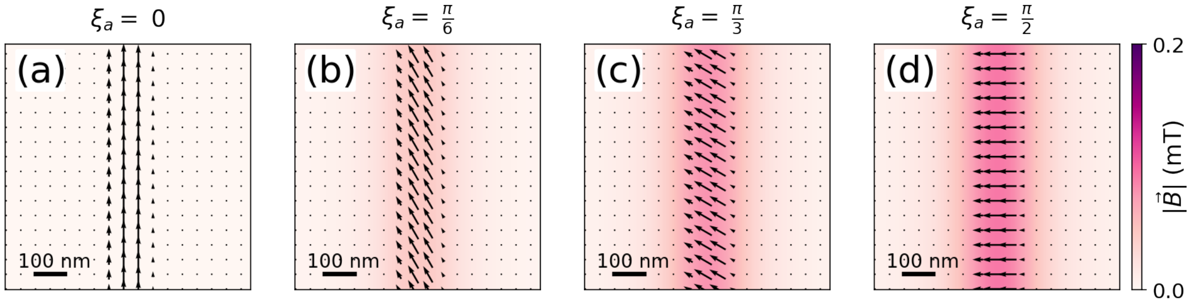

Figure S8: Field magnitude of ADWs with different phase ξ_a. Field magnitude $|\vec{B}|$ obtained from the magnetic field calculations of ADW models with different ξ_a. Black arrows illustrate $\vec{m}_{xy}$.

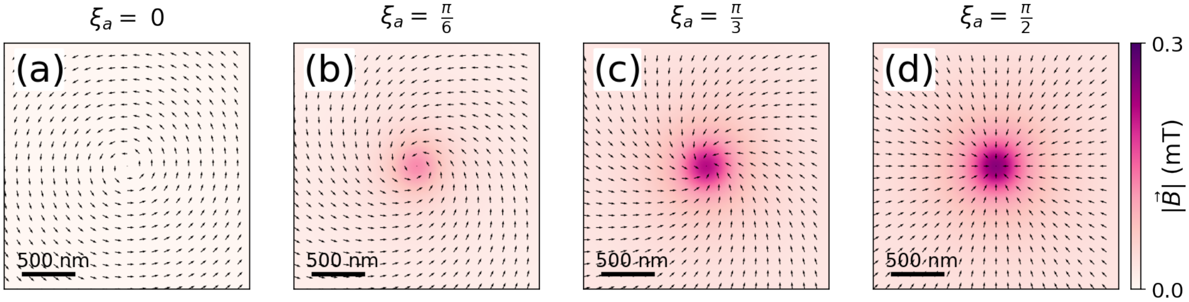

Figure S9: Magnetic field magnitude of merons with different phase ξ_a. Field magnitude $|\vec{B}|$ obtained from the magnetic field calculations of meron models with different ξ_a. Black arrows illustrate $\vec{m}_{xy}$.

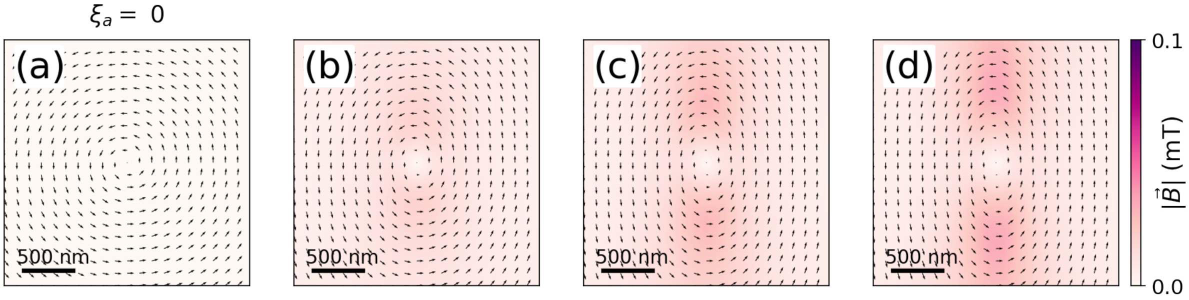

Figure S10: Distortion of a divergence-free texture. Field magnitude $|\vec{B}|$ exhibited by an a-Néel meron with increasing distortion to the surrounding $\vec{m}_{xy}$ distribution from (a) to (d). Black arrows illustrate $\vec{m}_{xy}$.

S5.7 Magnetic Field and Charge of AFM Bimerons

An AFM bimeron in a uniform $\vec{m}_{xy}$ background can be described analytically as a skyrmion model rotated by 90° around an in-plane axis. Assuming a phase ξ_a and radial size R_B, we have:

$$\vec{l} = 2\ M_s[\ \cos\theta,\ \sin\theta\sin(\phi+\xi_a),\ -\sin\theta\cos(\phi+\xi_a)]\ , \tag{S17}$$

$$\vec{m} = 2\Delta M_s\ [-\ \sin\theta\sin(\phi+\xi_a),\ \cos\theta,\ 0\ ]\ , \tag{S18}$$

$$\text{where, } \theta = \begin{cases} \frac{\pi r}{R_B} & , r \leq R_B \\ \pi & , r > R_B \end{cases} .$$

Hence,

$$\vec{\nabla} \cdot \vec{m}_{xy} = \begin{cases} 2\Delta M_s \big[-\frac{\pi}{R_B} \big(\cos\theta \cos\phi \sin(\phi + \xi_a) + \sin\theta \sin\phi \big) \\ \qquad\qquad + \frac{1}{r} \sin\theta \sin\phi \cos(\phi + \xi_a) \big] & , r \leq R_B \\ 0 & , r > R_B \end{cases} . \tag{S19}$$

Equation S19 allows us to model the σ_m distribution of bimerons, see Fig. S11. Here, we observe that individual monopolar and quadrupolar σ_m distributions contributed by the meron and antimeron components, albeit significantly distorted in comparison to their isolated counterparts, can be discerned in the σ_m distribution of an isolated AFM bimeron. This is slightly more evident in the case of a-Bloch bimerons ($\xi_a = \pi/2$, $3\pi/2$). For a-Néel bimerons the σ_m distribution only reflects a strongly distorted quadrupolar character as the meron component is relatively divergence-free.

More importantly, we observe a non-zero σ_m distribution, irrespective of the ξ_a value, implying that bimerons of all characters generate distinct and detectable B_z signatures, see Fig. S12. This enables DQM to play a versatile role in the future discovery and study of isolated AFM bimerons in engineered AFM systems with a defined in-plane anisotropy and inhomogeneous DMI. Interestingly, while the integrated charge Q_m over a sufficiently large area centred at the core of the bimeron vanishes for all ξ_a, the asymmetric σ_m distribution suggests that it may be possible to spatially manipulate AFM bimerons via external magnetic fields.

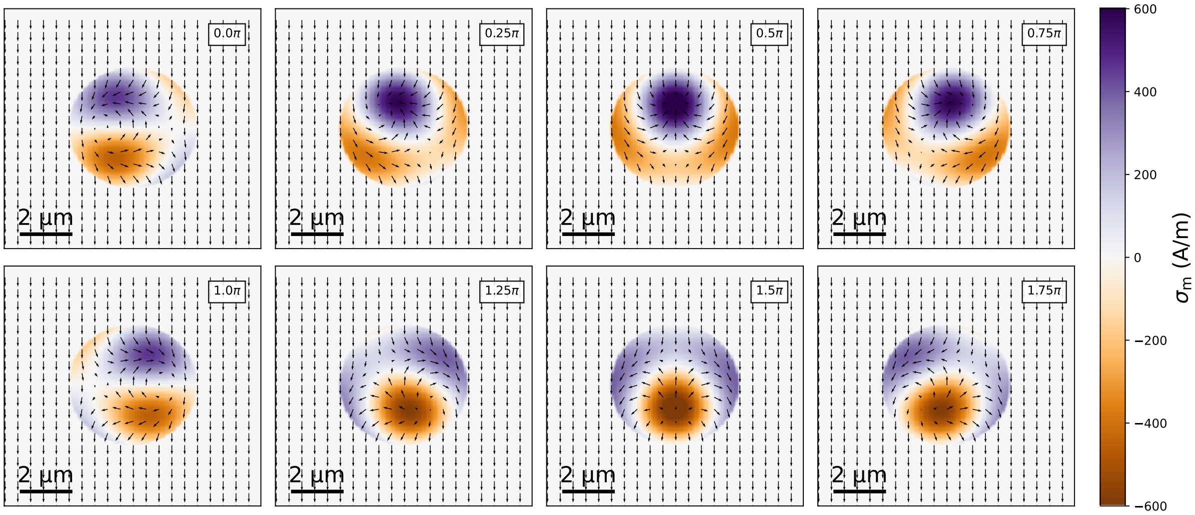

Figure S11: Magnetic charge density σ_m of an AFM bimeron with varying phase ξ_a. Black arrows illustrate $\vec{m}_{xy}$.

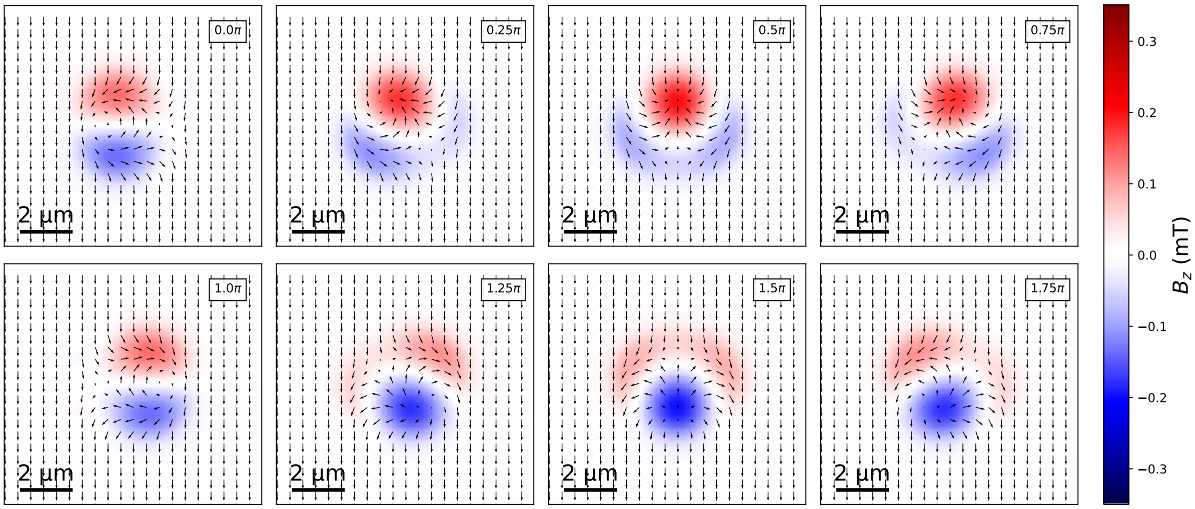

Figure S12: Magnetic field distribution of an AFM bimeron with varying phase ξ_a. Black arrows illustrate $\vec{m}_{xy}$.

S5.8 Magnetic Field and Charge of AFM Skyrmions

We can adapt the treatment from the previous section to construct the magnetic distribution in AFM skyrmions. Assuming a phase ξ_a and radial size R_S, we have:

$$\vec{l} = 2\ M_s[\ \sin\theta\cos(\phi+\xi_a),\ \ \sin\theta\sin(\phi+\xi_a),\ \ \cos\theta]\ , \tag{S20}$$

$$\vec{m} = 2\Delta M_s[\ -\sin\theta\sin(\phi+\xi_a),\ \ \sin\theta\cos(\phi+\xi_a),\ 0]\ , \tag{S21}$$

$$\text{where, } \theta = \begin{cases} \frac{\pi r}{R_S} & , r \leq R_S \\ \pi & , r > R_S \end{cases} .$$

Hence,

$$\vec{\nabla}\cdot\vec{m}_{xy} = \begin{cases} 2\Delta M_s \sin\xi_a\left(\frac{-\sin\theta}{r} - \frac{\pi\cos\theta}{R_S}\right) & , r \leq R_S \\ 0 & , r > R_S \end{cases} . \tag{S22}$$

AFM Néel skyrmions ($\xi_a = 0, \pi$) have vanishing σ_m, as they host divergence-free $\vec{m}_{xy}$. Other skyrmions with $\xi_a \neq 0, \pi$ have a non-zero σ_m distribution, see Fig. S13. As expected, B_z mirrors the σ_m trend, Fig. S14. While AFM Néel skyrmions are invisible, DQM can still play a crucial role in imaging AFM skyrmions with mixed or a-Bloch characters, with the added advantage of reading out the projected vorticity via the strength of B_z. In actuality, Néel skyrmions undergo deformation/distortion (*19*), which could result in detectable magnetic field signatures, akin to what is expected for distorted a-Néel merons, as illustrated in Fig. S10.

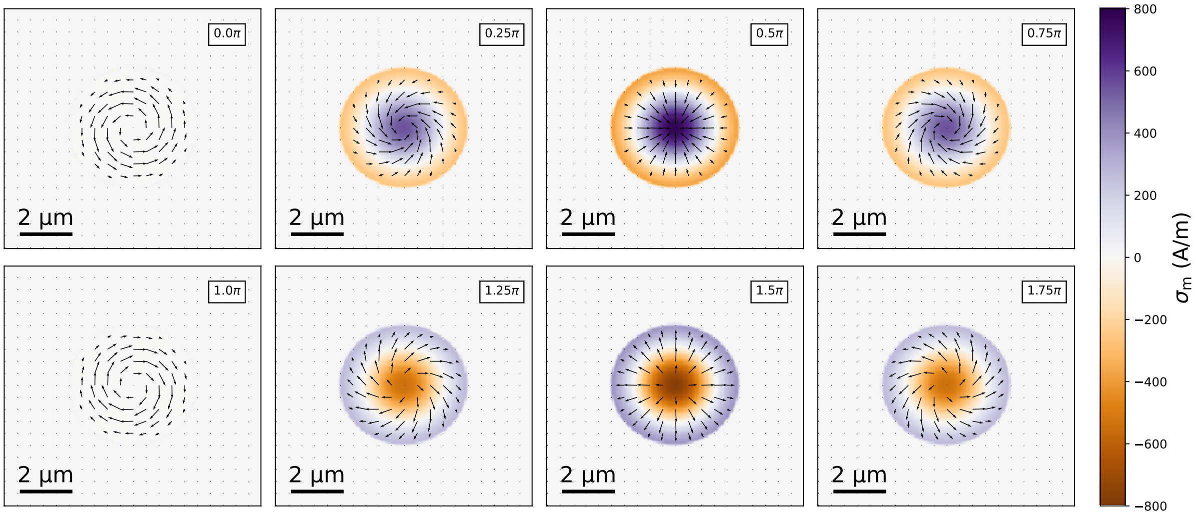

Figure S13: Magnetic charge density σ_m distribution of an AFM skyrmion with varying phase ξ_a. Black arrows illustrate $\vec{m}_{xy}$.

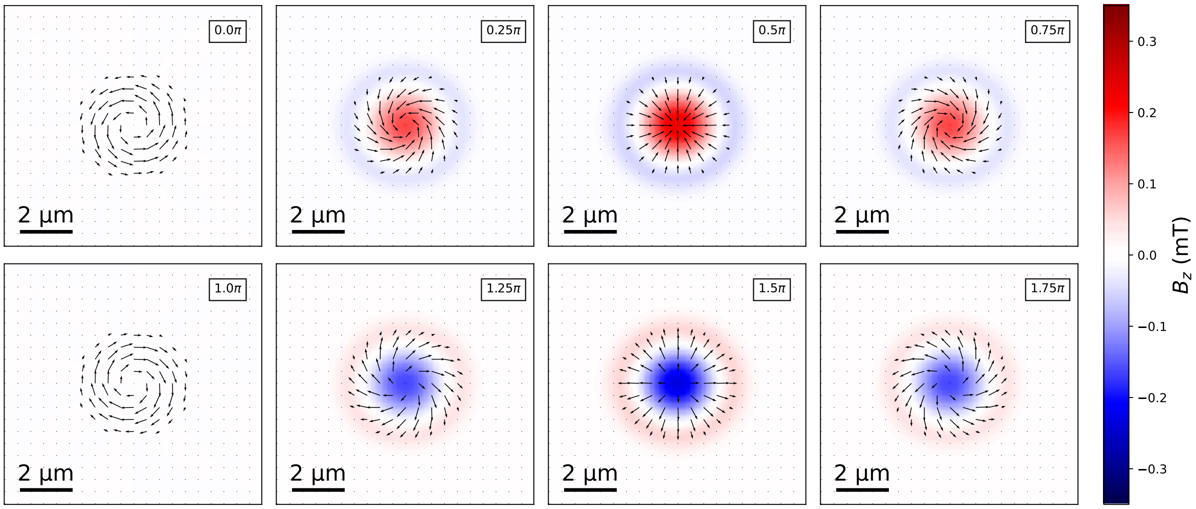

Figure S14: Magnetic field distribution of an AFM skyrmion with varying phase ξ_a. Black arrows illustrate $\vec{m}_{xy}$.

S5.9 Integrated Magnetic Charge of AFM Merons and Antimerons

The integrated charge Q_m of an isolated (anti)meron with phase ξ_a and winding number $\mathcal{N}$ as a function of radial distance r from the core can be analytically written as:

$$Q_m(r) = \begin{cases} 4\pi M_s\,\Delta\,\sin(\xi_a)\,\sin\left(\frac{\pi r}{2R_M}\right) r\,t & , \; r \leq R_M, \; \mathcal{N} = +1 \\ 4\pi M_s\,\Delta\,\sin(\xi_a)\, r\,t & , \; r > R_M, \; \mathcal{N} = +1 \\ 0 & , \mathcal{N} = -1 \end{cases} \cdot \quad \text{(S23)}$$

For isolated symmetric textures, Q_m is either zero for antimerons ($\mathcal{N} = -1$) and a-Néel merons ($\mathcal{N} = +1$, $\xi_a = 0, \pi$), or accumulates radially ($\mathcal{N} = +1, \xi_a \neq 0, \pi$), as illustrated in Fig. S15(d).

Moreover, the integrated charge Q_m of an isolated bimeron with phase ξ_a can be written as:

$$Q_m(r) = \begin{cases} 2\pi M_s\,\Delta\,\sin(\xi_a)\,\sin\left(\frac{\pi r}{R_B}\right) r\,t & , \; r \leq R_B \\ 0 & , \; r > R_B \end{cases} . \quad \text{(S24)}$$

While isolated bimerons with $\xi_a \neq 0, \pi$ accumulate some charge at short distances near their cores, see Fig. S15(d), the total integrated charge, outside R_B, for all isolated bimerons embedded in a uniformly aligned magnetic background is identically zero.

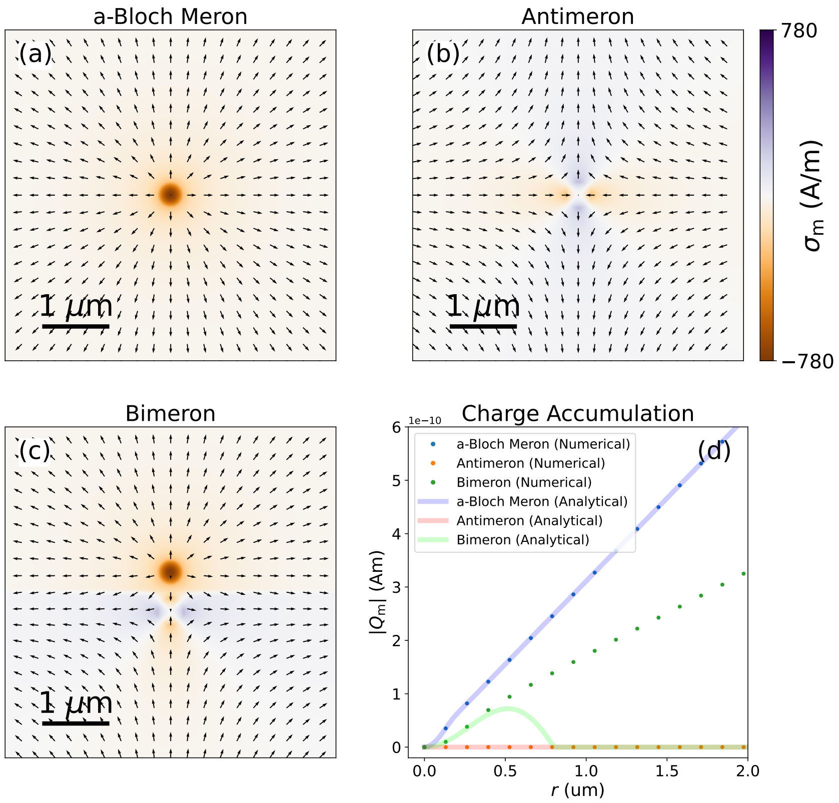

Figure S15: Charge accumulation of isolated textures. Numerical simulation of charge density σ_m distribution of (a) an a-Bloch meron, (b) an antimeron and (c) a bimeron. Arrows represent $\vec{m}_{xy}$. (d) The total charge $|Q_m|$ accumulated in a circular area S of increasing radius r, for each numerical simulation in (a-c), is given by the solid dots. Q_m from analytical equations S23 and S24 are represented by solid curves. The analytical Q_m for a a-Bloch meron and an antimeron ansatz agree well with the numerically obtained Q_m.

S5.10 Charge Variability of AFM Bimerons

The total charge Q_m of a bimeron in a fixed area S can be varied with different $\vec{m}_{xy}$ boundary conditions. Figs. S16(a-e) illustrate bimerons with various boundary conditions, alongside their $\vec{m}_{xy}$ and σ_m distributions. Figure S15(f) shows the variability of $|Q_m|$ corresponding to panels (a-e), where each $|Q_m|$ is obtained from the integration of their respective σ_m distribution over the whole region shown. Notably, in panel (e), the bimeron is embedded in a uniform magnetisation, similar to the analytical a-Bloch bimeron charge distribution in Fig. S11, with a

boundary condition that satisfies:

$$Q_m = \int_S \sigma_m \, ds \propto \oint_C \vec{m}_{xy} \cdot \hat{e} \, dc = 0, \tag{S25}$$

where C is the boundary of the area of integration S and $\hat{e}$ is the unit normal to C. Figure S16(e) qualitatively recovers the analytical charge distribution of an a-Bloch bimeron in Fig. S11 including producing zero total charge Q_m, with a non-zero charge distribution embedded in a uniform and divergence-free magnetisation. Nonetheless, both charge distributions appear contrasting mainly due to differences in the distance between meron and antimeron, and the shape of the bimeron as outlined by the $\vec{m}_{xy}$ neighborhood which is circular for the analytical case (Fig. S11) and rectangle for the numerical case (Fig. S16).

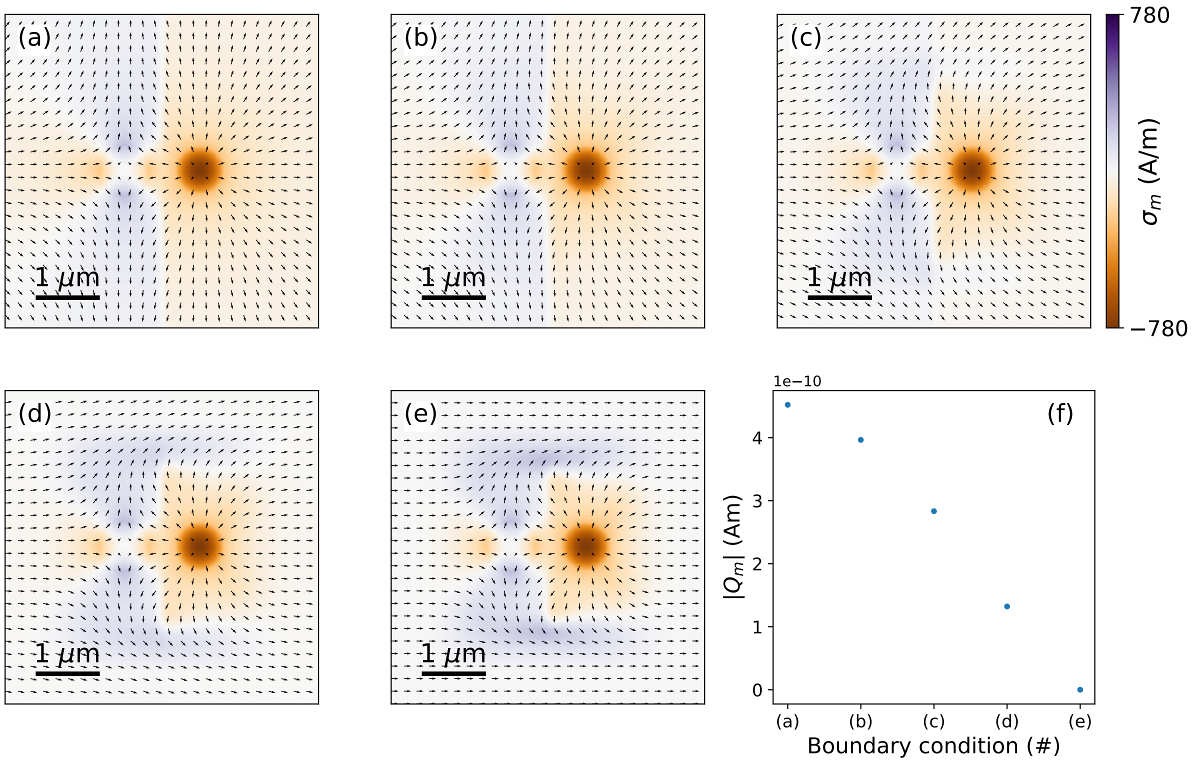

Figure S16: Charge variability of a bimeron. (a-e) Numerical calculations of charge density σ_m distribution of bimerons with various $\vec{m}_{xy}$ boundary conditions. Arrows represent $\vec{m}_{xy}$. (f) Total charge $|Q_m|$ accumulated in a fixed area S of various bimeron simulation with different $\vec{m}_{xy}$ boundary conditions in (a-e). For convenience, S is defined as the square area covering the entirety of each panel, and is centered at the midpoint between the core of the meron and the antimeron.

S5.11 Topological Numbers

Three-dimensional AFM textures can be characterised by two topological numbers (*20*):

i The topological charge, Q, is defined as an area integral:

$$Q = \frac{1}{4\pi}\int \hat{l}\cdot\left(\frac{\partial \hat{l}}{\partial x}\times\frac{\partial \hat{l}}{\partial y}\right) dA,$$

where A is the area and $\hat{l} = \frac{\vec{l}}{|\vec{l}|}$. Q is $\pm 1/2$ for AFM (anti)meron, and ± 1 for bimerons and skyrmions.

ii The topological winding number, $\mathcal{N}$, is defined as a close line integral of the azimuthal, ϕ, change of the Néel vector, $\vec{l}$, around the topological core:

$$\mathcal{N} = \frac{1}{2\pi} \oint d\phi.$$

$\mathcal{N}$ is $+1$ for AFM merons and skyrmions, and -1 for AFM antimerons and antiskyrmions.

S6 Meron Core Size Analysis

The experimental $\vec{m}_{xy}$ reconstruction of merons and antimerons (see S7) requires an estimation of the meron core size. Here, the average meron radial profile shown in Fig. S17 is obtained from the merons found in main text Fig. 1(h), by averaging their azimuthally–averaged radial B_z profiles. In general, there is good qualitative agreement with the calculated B_z profiles obtained from the structures in section S5. Any quantitative deviations are expected to result from the limitations of the linear meron ansatz and of a comparison between experimental data obtained from a composite textural fabric and an isolated meron model. We estimate $R_M \sim 150 \pm 40$ nm based on the maximum value of B_z, which corresponds reasonably well with our previous work (*1*). Addressing the deviations in and around the meron core requires theoretical refinement of the meron model alongside shallow NV imaging.

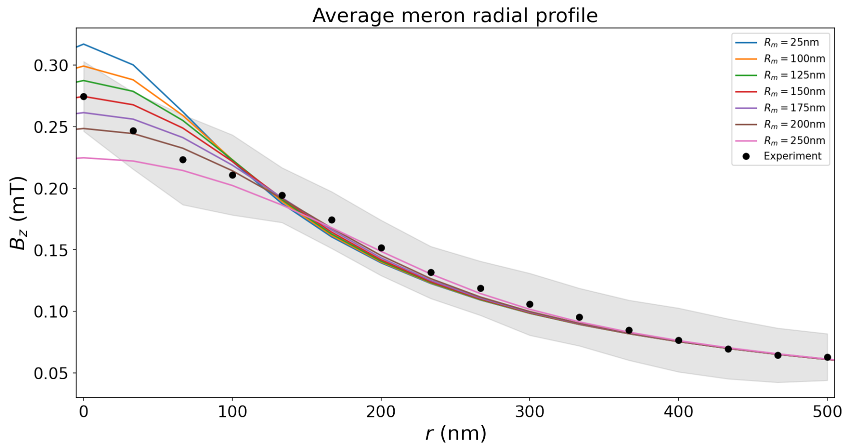

Figure S17: Average meron core size. The average experimental B_z profile of six merons as a function of radial distance r from the center of the meron core (solid black dots, shaded area is the standard deviation). The experimental profile is compared with the calculated B_z profiles of meron models with various core sizes R_M (coloured lines).

S7 Retrieving $\vec{m}_{xy}$

The inverse magnetostatic problem of retrieving $\vec{m}$ from $\vec{B}$ is in general ill posed. However, in our case, we can take advantage of existing constrains to approximate a solution. We first simplify the problem, as discussed in section S7.1, by utilizing the fact that the magnetic field components are linearly dependent, and that the out-of-plane magnetisation component vanishes in α-Fe_2O_3. Next in section S7.2, we adopt appropriate regularisation strategies to obtain $\vec{m}_{xy}$ from B_z. Finally, some examples are discussed in sections S7.3 and S7.4. Note that the antiferromagnetic vorticity and magnetic charge presented in this work are independent of our magnetisation retrieval protocols. Both aspects are based on the measured B_z as detailed in equation 1 and 2 in the main text.

S7.1 Simplification of Retrieval Problem

Retrieving $\vec{m}$ from $\vec{B}$ at distance d above the sample surface (i.e., inversion of Eqn. S1) is an ill-posed problem and is therefore not uniquely solvable. The problem can be simplified to one field component, since the other two are linearly dependent on the first one. We chose to work with B_z since it intuitively reveals a low-pass filtered $\vec{\nabla} \cdot \vec{m}_{xy}$ (eqn. S3). The inverse of the map $\boldsymbol{f}$ such that $B_z = \boldsymbol{f}(\vec{m})$, is not still not unique, even within the restricted class of problems represented by the dipolar tensor (eqn. S1). However, a choice of appropriate constraints reduces the degrees of freedom, thereby allowing us to estimate $\vec{m}$ from a DQM scan. In the present case, we set the out-of-plane magnetisation component $m_z = 0$. The inversion problem is thus reduced from three to two dimensions, but the reduced dimensionality is still not sufficient to estimate the in-plane magnetisation vector. This is clear when considering equation S2, where we show that any divergence-free $\vec{m}_{xy}$, including trivial ordering, produces the same field distribution ($|B| = 0$, section S5.6).

S7.2 Regularisation

We adopt several regularisation strategies developed by the Lorentz transmission electron microscopy community to approximate a solution. Closely following the strategy in (*21*), we replace the ill-posed inversion problem with the following minimisation problem:

$$\min \left(\|\mathbf{Fm} - \mathbf{B}_z\|^2 + \|\mathbf{\Gamma m}\|^2 \right) \ , \tag{S26}$$

where, $\mathbf{F}$, $\mathbf{B}_z$ and $\mathbf{m}$ are the matrix representation of the map $\boldsymbol{f}$, the observed B_z and the reconstruction of $\vec{m}$, respectively. The spatial discretisation, implicit to the minimisation equation and also to a DQM scan, reduces the problem to finite dimensions and limits the maximum resolvable signal frequency to the inverse of the spatial resolution (i.e. $< \frac{1}{33}$ GHz in our im-

ages). The first term in equation S26 is the squared error needed for the base minimisation. The second term, known as the Tikhonov regularisation, is used to impose a smoothness constraint to the solution $\mathbf{m}$. This is achieved via $\mathbf{\Gamma m}$ where $\mathbf{\Gamma} = \lambda\nabla$ is the matrix representation of the gradient ∇ weighted by the constant λ. We constrain the problem further by fixing the magnitude of the canted magnetisation $|\vec{m}_{xy}|$. Note that $|\vec{m}_{xy}|$ in real space varies across a domain wall, or a (anti)meron core following a linear model (section S5). Given that $\vec{m}_{xy}$ can be written as $|\vec{m}_{xy}|(\cos\phi_m, \sin\phi_m)$, one only needs to reconstruct the angle ϕ_m. However, the reduction of the problem to one dimension alone does not guarantee a unique solution as we still have a non-trivial null space. Finally, to quicken the minimisation and to avoid local minima, we initialise the problem with a $\mathbf{m}$ guess based on *a priori* information of our system (*1*). ϕ_m is initialised based on the expected topological texture associated with the characteristic B_z distribution shown analytically and via modeling in Fig. 2 in the main text, whereas $|\vec{m}_{xy}|$ is fixed based on the linear meron ansatz (section S5) with an average core size of 150 nm (section S6), and the linear ADW ansatz with a similar domain wall width (section S10). In this respect, we only attempt to reconstruct $\vec{m}_{xy}$ for AFM textures and their immediate neighbourhood with characteristic B_z signatures. Additionally, we minimise the problem with B_z at $z = d_{NV}$ rather than $z = 0$ to avoid using low pass filters for the propagation, and with Neumann boundary condition to reduce edge effects.

S7.3 Fitting of Antiphase Domain Walls

We assume $|\vec{m}_{xy}| = 0$ outside the ADW. Inside the ADW, $|\vec{m}_{xy}|$ varies according to the linear ADW model (section S10). It is easy to separate these areas since out-of-plane AFM domains have negligible divergence while the ADWs have a distinct B_z signature. We show an example of the reconstruction process from the B_z image of a model multi-chiral ADW $\vec{m}_{xy}$ distribution in Fig. S18(a). The $|\vec{m}_{xy}|$ mask is obtained by estimating the binary mask from the calculated

B_z, and then modulating it as per the linear ansatz, see Fig. S18(b). Here we initialise the problem with a trivial $\phi_m(x, y) = 0$ and the corresponding $\vec{m}_{xy}$ guess is given in Fig. S18(c). The minimised $\vec{m}_{xy}$ solution given in Fig. S18(d) faithfully reproduces the modelled $\vec{m}_{xy}$ distribution, with errors localised at the edges as indicated by the fit residues in Figs. S18(e) and (f). Next, we apply the same protocol to the experimentally obtained B_z, with its respective $w \sim 2R_m$ and minimised $\vec{m}_{xy}$ solution given in Figs. S19.

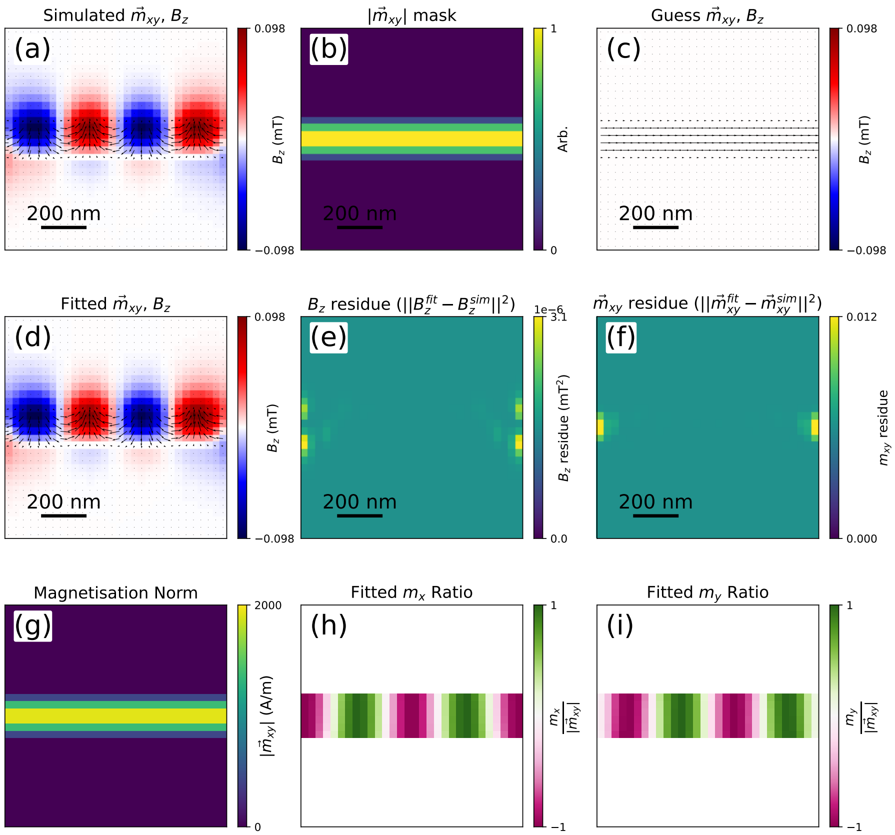

Figure S18: Model ADW fit. (a) modelled $\vec{m}_{xy}$ of an AFM ADW and the resultant B_z at $d_{NV} = 70$ nm. **(b-c)** (b) Fixed $|\vec{m}_{xy}|$ mask and (c) guess $\vec{m}_{xy}$ distribution used to initialise the minimisation problem. **(d-f)** (d) Minimised $\vec{m}_{xy}$ solution and respective (e) B_z and (f) $\vec{m}_{xy}$ residues. Black arrows illustrate $\vec{m}_{xy}$. **(g-i)** (g) Magnetisation norm, (h) ratio of m_x to $|\vec{m}_{xy}|$, and (i) ratio of m_y to $|\vec{m}_{xy}|$.

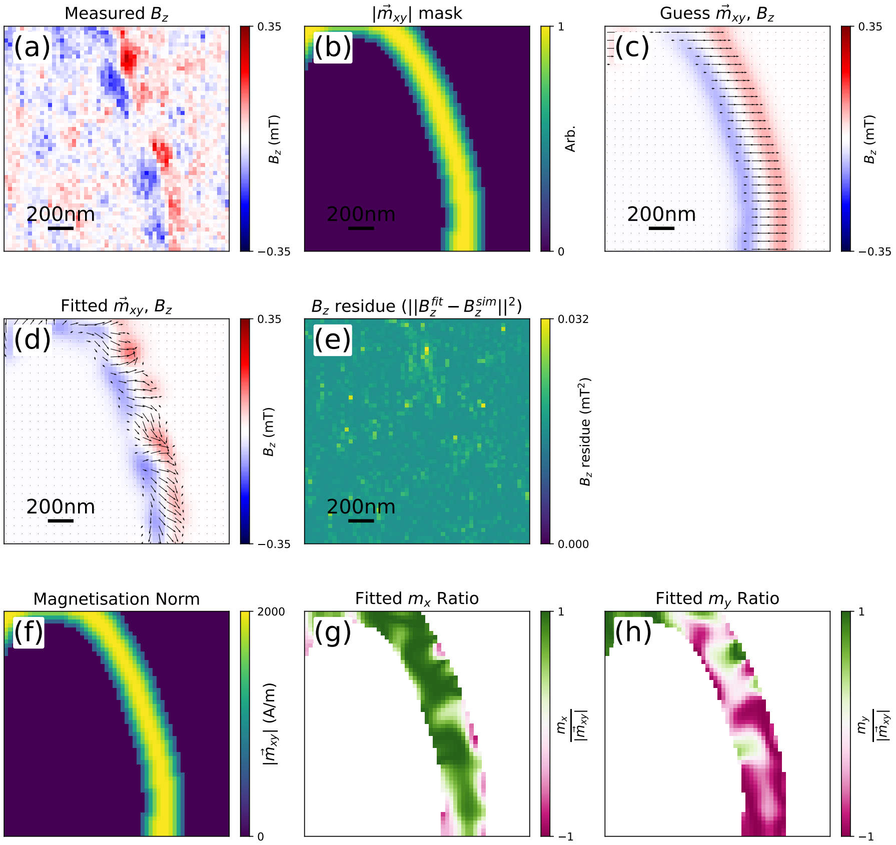

Figure S19: Experimental ADW fit. (a) Measured B_z of an AFM ADW at $d_{NV} = 70$ nm. **(b-c)** (b) Fixed $|\vec{m}_{xy}|$ mask and (c) guess $\vec{m}_{xy}$ distribution used to initialise the minimisation problem. **(d-e)** (d) Minimised $\vec{m}_{xy}$ solution and respective (e) B_z residue. The image is taken at $T \sim 5$ K. Black arrows illustrate $\vec{m}_{xy}$. **(f-h)** (f) Magnetisation norm, (g) ratio of m$_x$ to $|\vec{m}_{xy}|$, and (h) ratio of m$_y$ to $|\vec{m}_{xy}|$.

S7.4 Fitting of (Anti)Merons and Bimerons

Similar to the procedure in section S7.3, $|\vec{m}_{xy}| = 1$ is assumed everywhere except in the (anti)meron core, which is modelled with a linear ansatz with $R_M = 150$ nm (section S6). We show the $\vec{m}_{xy}$ reconstruction of a model a-Bloch AFM meron and an AFM antimeron in Figs. S20 and S21, respectively. In both cases, we start off with ξ_a that deviates from the model texture, and the corresponding $\vec{m}_{xy}$ guess is given in panel (c). The resultant minimised solution given in panel (d) closely reproduces the model $\vec{m}_{xy}$ distribution in panel (a). The respective

residues are given in panels (e-f).

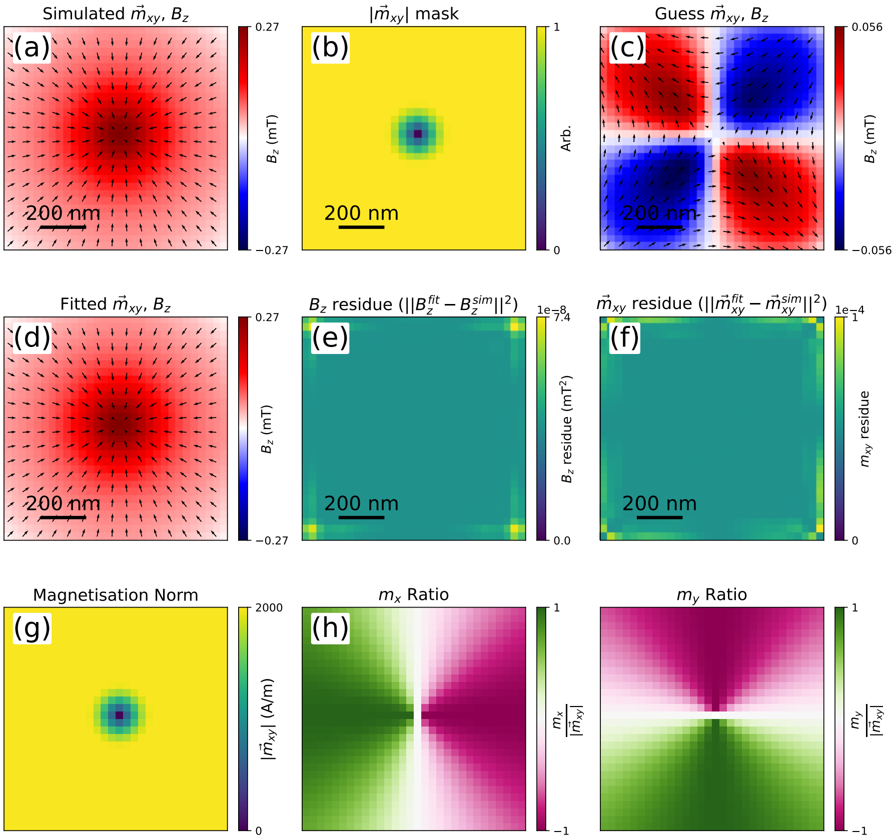

Figure S20: Model a-Bloch meron fit. modelled $\vec{m}_{xy}$ of an AFM a-Bloch meron and the resultant B_z at $d_{NV} = 70$ nm. **(b-c)** (b) Fixed $|\vec{m}_{xy}|$ mask and (c) guess $\vec{m}_{xy}$ distribution used to initialise the minimisation problem. **(d-f)** (d) Minimised $\vec{m}_{xy}$ solution and respective (e) B_z and (f) $\vec{m}_{xy}$ residues. Black arrows illustrate $\vec{m}_{xy}$. **(g-i)** (g) Magnetisation norm, (h) ratio of m$_x$ to $|\vec{m}_{xy}|$, and (i) ratio of m$_y$ to $|\vec{m}_{xy}|$.

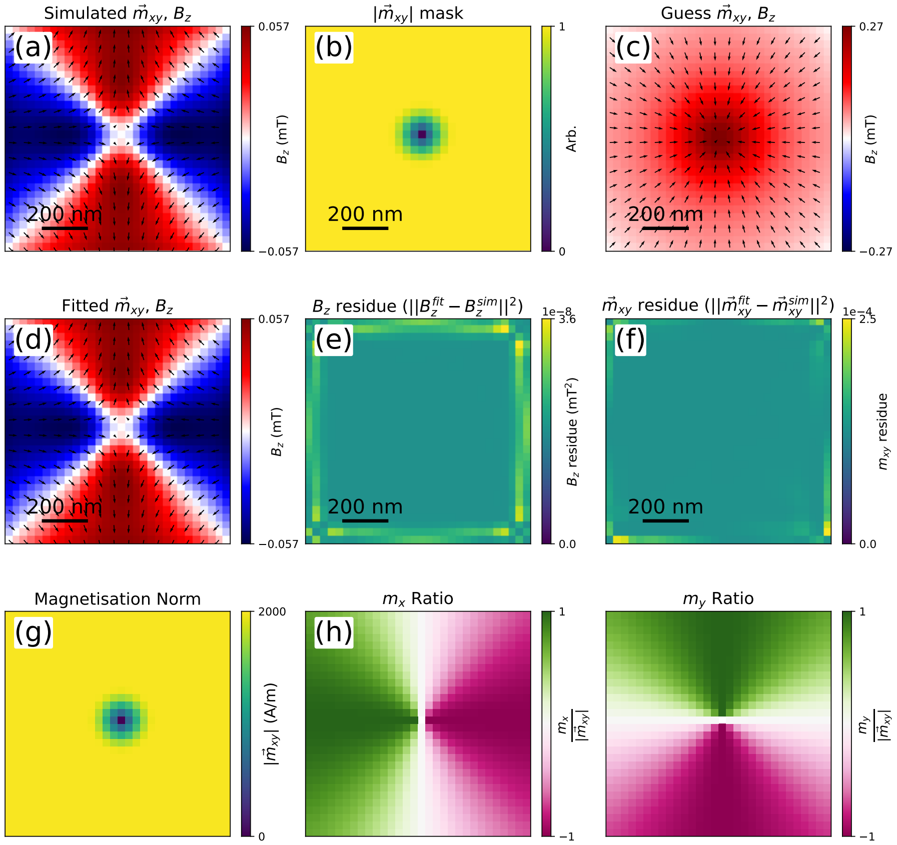

Figure S21: Model antimeron fit. modelled $\vec{m}_{xy}$ of an AFM antimeron and the resultant B_z at $d_{NV} =$ 70 nm. **(b-c)** (b) Fixed $|\vec{m}_{xy}|$ mask and (c) guess $\vec{m}_{xy}$ distribution used to initialise the minimisation problem. **(d-f)** (d) Minimised $\vec{m}_{xy}$ solution and respective (e) B_z and (f) $\vec{m}_{xy}$ residues. Black arrows illustrate $\vec{m}_{xy}$. **(g-i)** (g) Magnetisation norm, (h) ratio of m$_x$ to $|\vec{m}_{xy}|$, and (i) ratio of m$_y$ to $|\vec{m}_{xy}|$.

Finally, we apply the same reconstruction protocol to experimental images, from which we obtain the $\vec{m}_{xy}$ fits of an anti-clockwise a-Bloch meron (Fig. S22), an antimeron (Figure S23) and a composite structure consisting of one meron and two antimerons (Fig. S24).

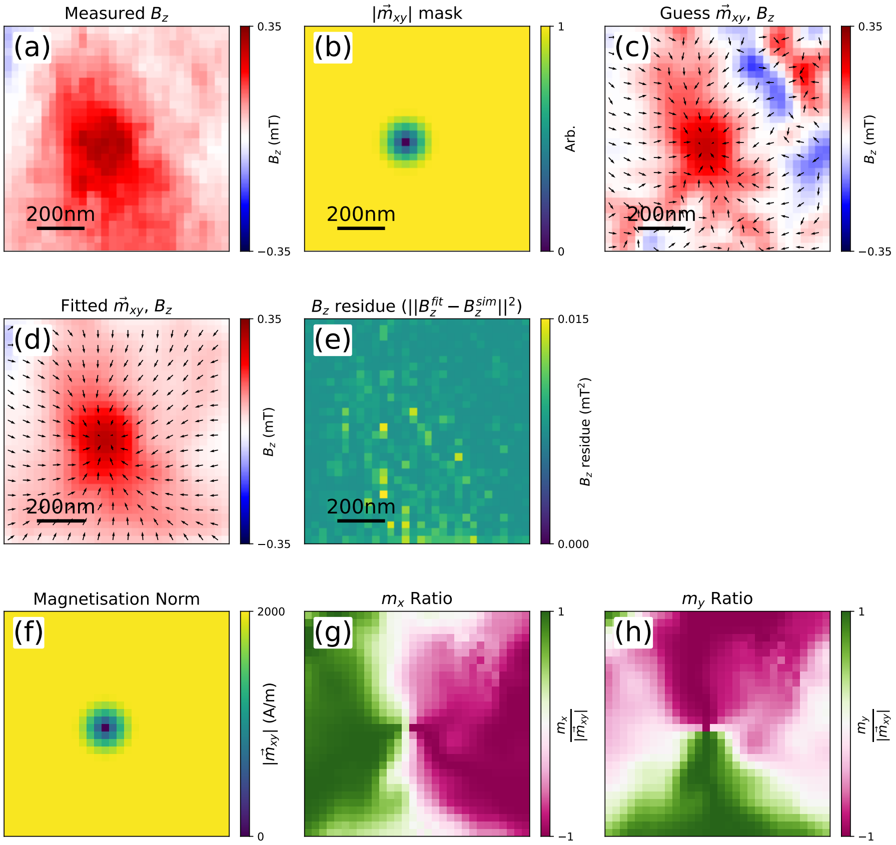

Figure S22: Experimental a-Bloch meron fit. Measured B_z of an AFM anti-clockwise a-Bloch meron at $d_{NV} = 70$ nm. **(b-c)** (b) Fixed $|\vec{m}_{xy}|$ mask and (c) guess $\vec{m}_{xy}$ distribution used to initialise the minimisation problem. **(d-e)** (d) Minimised $\vec{m}_{xy}$ solution and respective (e) B_z residue. Black arrows illustrate $\vec{m}_{xy}$. **(f-h)** (f) Magnetisation norm, (g) ratio of m$_x$ to $|\vec{m}_{xy}|$, and (h) ratio of m$_y$ to $|\vec{m}_{xy}|$.

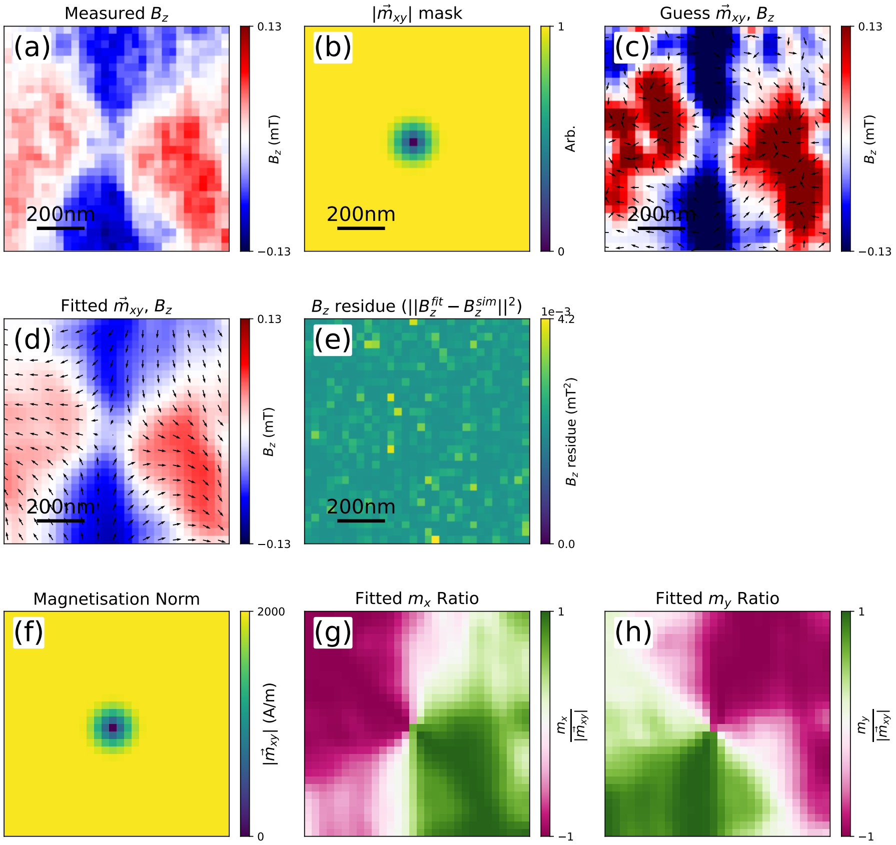

Figure S23: Experimental antimeron fit. Measured B_z of an AFM antimeron at $d_{NV} = 70$ nm. **(b-c)** (b) Fixed $|\vec{m}_{xy}|$ mask and (c) guess $\vec{m}_{xy}$ distribution used to initialise the minimisation problem. **(d-e)** (d) Minimised $\vec{m}_{xy}$ solution and respective (e) B_z residue. Black arrows illustrate $\vec{m}_{xy}$. **(f-h)** (f) Magnetisation norm, (g) ratio of m$_x$ to $|\vec{m}_{xy}|$, and (h) ratio of m$_y$ to $|\vec{m}_{xy}|$

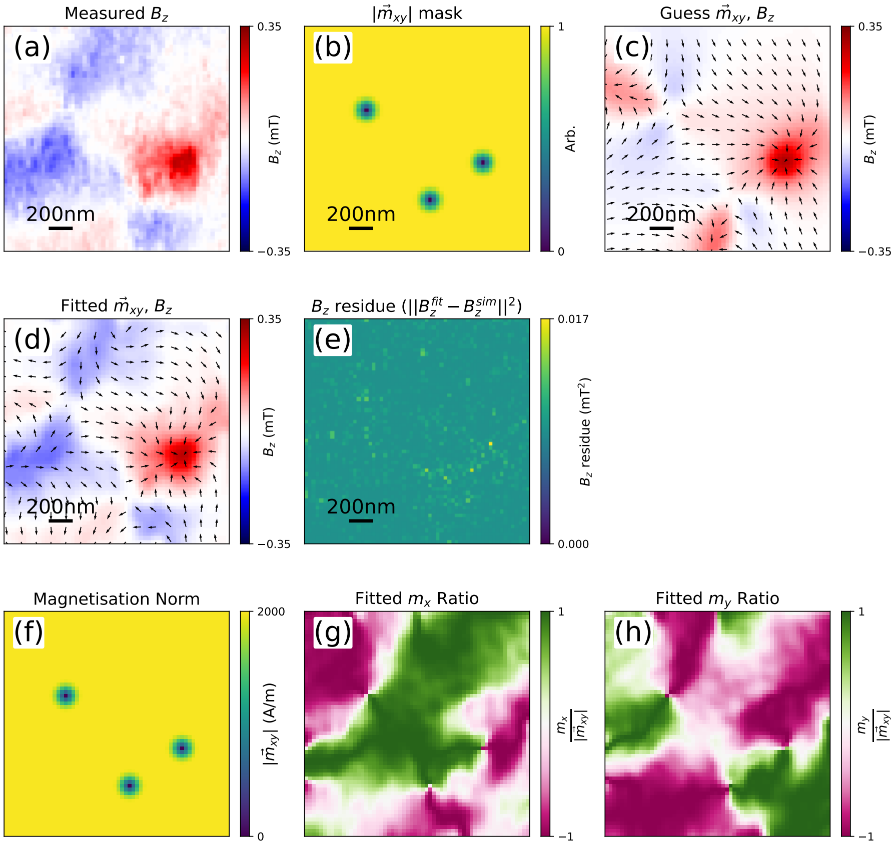

Figure S24: Experimental fit for composite texture. (a) Measured B_z of a composite texture consisting of two antimerons and an a-Bloch meron at $d_{NV} = 70$ nm. **(b-c)** (b) Fixed $|\vec{m}_{xy}|$ mask and (c) guess $\vec{m}_{xy}$ distribution used to initialise the minimisation problem. **(d-e)** (d) Minimised $\vec{m}_{xy}$ solution and respective (e) B_z residue. Black arrows illustrate $\vec{m}_{xy}$. **(f-h)** (f) Magnetisation norm, (g) ratio of m$_x$ to $|\vec{m}_{xy}|$, and (h) ratio of m$_y$ to $|\vec{m}_{xy}|$.

S7.5 Fitting Multiple Merons and Antimerons

Weighted Model. For the fitting of multiple meron/antimeron in a single window, we adopt a weighted model to combine the linear ansatz $\vec{m}^{(n)}$ of each individual texture n to generate a guess solution that includes a number p of structures. Note that we start with a coreless meron/antimeron first (i.e. $R_M = 0$) but will account for the core size after mixing. The magnetisation $\vec{m}$ of a meron and an antimeron is given by equation S13, and should account for

the core position $\vec{r_0}^{(n)} = \left(x_0^{(n)}, y_0^{(n)}\right)$ such that $\vec{m}^{(n)}(\vec{r}) = \vec{m}(\vec{r} - \vec{r_0}^{(n)})$ and $\vec{r} = (x, y)$ represents the cartesian coordinates. The guess solution is generated by a weighted model given by:

$$\vec{m}_{\text{multi}}(\vec{r}) = \frac{\sum_{n=1}^{p} \vec{m}^{(n)}(\vec{r}) \cdot W^{(n)}(\vec{r})}{\sum_{n=1}^{p} W^{(n)}(\vec{r})}, \tag{S27}$$

where $W^{(n)}(\vec{r})$ is the weight associated to each meron/antimeron given by:

$$W^{(n)}(\vec{r}) = \frac{1}{|\vec{r} - \vec{r_0}^{(n)}| + c}. \tag{S28}$$

A small constant c is added to avoid a zero denominator. Here, c is simply the image resolution of $\sim$ 33 nm. Finally, we normalise the weighted magnetisation, insert the meron/antimeron cores and multiply it by the canted magnetisation:

$$\vec{m}_{\text{multi}}^{\text{guess}}(\vec{r}) = 2\Delta M_s \cdot \frac{\vec{m}_{\text{multi}}(\vec{r})}{|\vec{m}_{\text{multi}}(\vec{r})|} \cdot \prod_{n=1}^{p} \sin\theta^{(n)}, \tag{S29}$$

where $\sin\theta^{(n)}$ reduces the net magnetisation magnitude at the core of each meron/antimeron as per the linear ansatz (eqn. S13):

$$\theta^{(n)} = \begin{cases} \frac{\pi}{2R_M} \frac{|\vec{r} - \vec{r_0}^{(n)}|}{} & , |\vec{r}| < R_M \\ \frac{\pi}{2} & , |\vec{r}| > R_M \end{cases}.$$

Step-by-step Fitting Guide. A step-by-step fit of Fig. S24 is given here. We first identify the merons and antimerons via their characteristic Bz signature manually given by crosses as illustrated in Fig. S25. This can be done systematically since a meron and an antimeron exhibit distinct monopolar and quadrupolar distributions respectively (details in S5.5).

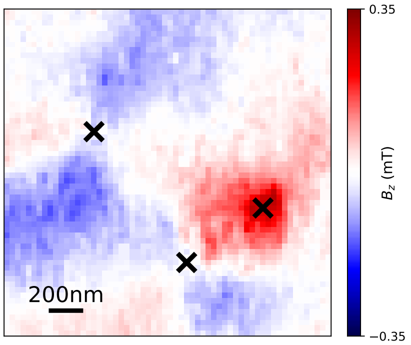

Figure S25: Identifying merons and antimerons. The positions of merons and antimerons are marked with crosses. Black arrows illustrate $\vec{m}_{xy}$.

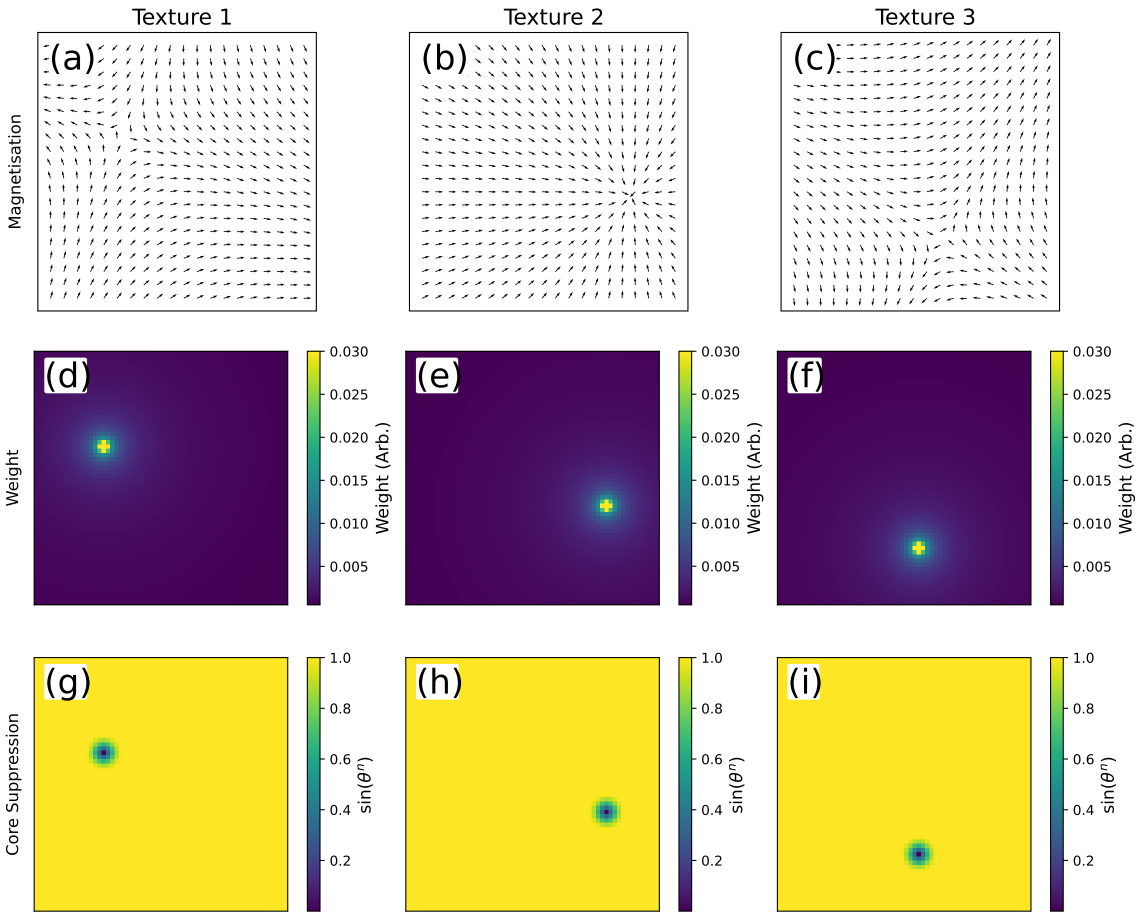

Figure S26: Step-by-step protocol to attaining a guess solution with multiple merons and antimerons. Magnetisation, weight and core suppression of individual meron and antimeron identified in Fig. S25. Black arrows illustrate $\vec{m}_{xy}$.

Next,we show the individual magnetisation $\vec{m}^{(n)}(\vec{r})$, weight $W^{(n)}(\vec{r})$ and core suppression $\sin\theta^{(n)}$ in Fig. S26. The guess solution $\vec{m}^{\text{guess}}_{\text{multi}}(\vec{r})$ following equations S27-S29 is given in Fig. S27 with the fit solution given in Fig. S24.

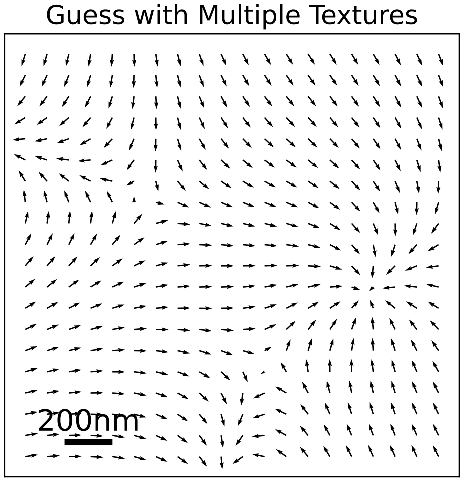

Figure S27: Weighted model guess solution. Final magnetisation guess based on the weighted model of individual meron and antimeron in Fig. S26. Black arrows illustrate $\vec{m}_{xy}$.

S7.6 Field Imaging and Magnetisation Reconstruction of α-Fe_2O_3

Unique magnetisation reconstruction is a general problem in the stray-field imaging. Nonetheless, the rigorous identification procedure (see S5.5), together with the implementation of systematic regularisation protocols, has helped constrain the magnetic solution space and provided meaningful canted moment reconstructions. While these reconstructions may not always be unique, they are in practice entirely consistent with AFM textures reported via X-ray imaging (*1*). A detailed discussion on the advantages of field imaging and the limitations of our reconstruction are given below.

Advantages of DQM imaging. On the one hand, whilst linear X-rays can locally resolve the staggered magnetisation or the AFM order ($\vec{l}$), there is a 180° ambiguity in the identification of its direction (*1, 17*) rendering it impossible to distinguish between clockwise and anticlockwise

a-Bloch merons. This issue does not affect DQM because the weak magnetic field is related to the *staggered vorticity*, $\vec{\mathcal{V}}$, of topological textures via the main text Equation 2. On the other hand, X-ray circular dichroism while sensitive to the canted magnetisation, $\vec{m}_{xy}$, were unsuccessful in our previous attempts to image topological AFM textures directly (*1*). The main reason is because in α-Fe_2O_3, the sublattice magnetisation has a canting angle of 1.1 mrad resulting in a $|\vec{m}_{xy}| \sim 10^3\mathrm{A/m}$ and in practice is too weak to produce circular dichroic X-ray contrast (*1*) (see S5). This issue does not hamper DQM because the NV center is extremely sensitive to weak magnetic fields emanating from the *weak* canted moment distributions. Therefore, the advantage of the NV over x-ray is its high sensitivity to weak magnetic fields and by extension to *weak* canted magnetisation in canted AFMs. It is due to this that field imaging via DQM provides an invaluable view of the rich magnetic charge phenomenology in α-Fe_2O_3.

Limitations of reconstruction. Provided $\vec{m}_{xy}$ or or its first derivative are known at the boundaries of the region of interest and that the magnitude $|\vec{m}_{xy}|$ is known everywhere, from the knowledge of $\vec{\nabla} \cdot \vec{m}_{xy}$ it is in principle possible to retrieve $\vec{m}_{xy}$ by solving Poisson's equation. However, for a sufficiently large area of interest, solutions exist in extremely narrow patches of phase space, which may be disjoint under certain conditions. Therefore, as a general strategy we have chosen to extract $\vec{m}_{xy}$by least-square minimisation starting from an initial guess, using Neumann's boundary conditions and a suitable ansatz for the spatial dependence of $|\vec{m}_{xy}|$. It must be understood that in many cases there will be residual ambiguity in the sign of the solenoidal part of $\vec{m}_{xy}$ (i.e., $\vec{\nabla} \cdot \vec{m}_{xy} = 0$).

For a mostly a-Bloch domain wall or meron, the solution will consistently give an a-Bloch solution with a small component of right or left a-Neel. An example of this is illustrated when fitting a domain wall with different initial conditions (Fig. S29), with each solution generally giving consistent a-Bloch component and an a-Neel component taking on two different and opposing directions (Fig. S30). Note that the a-Bloch component ($\vec{m}_{xy}$ parallel to the domain

wall) primarily contributes to the divergence while a-Neel ($\vec{m}_{xy}$ orthogonal to the domain wall) is responsible for the curl.

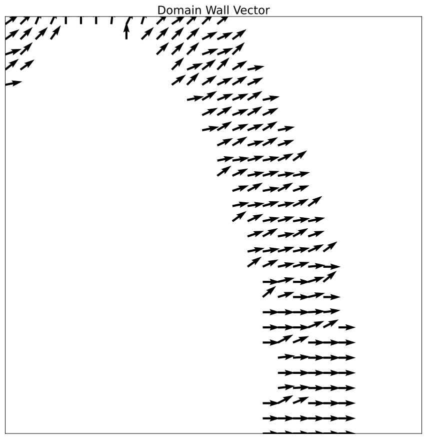

Figure S28: Domain wall vector. Arrows illustrate the domain wall vector retrieved from the experimental antiphase domain wall image in Fig. S19(a).

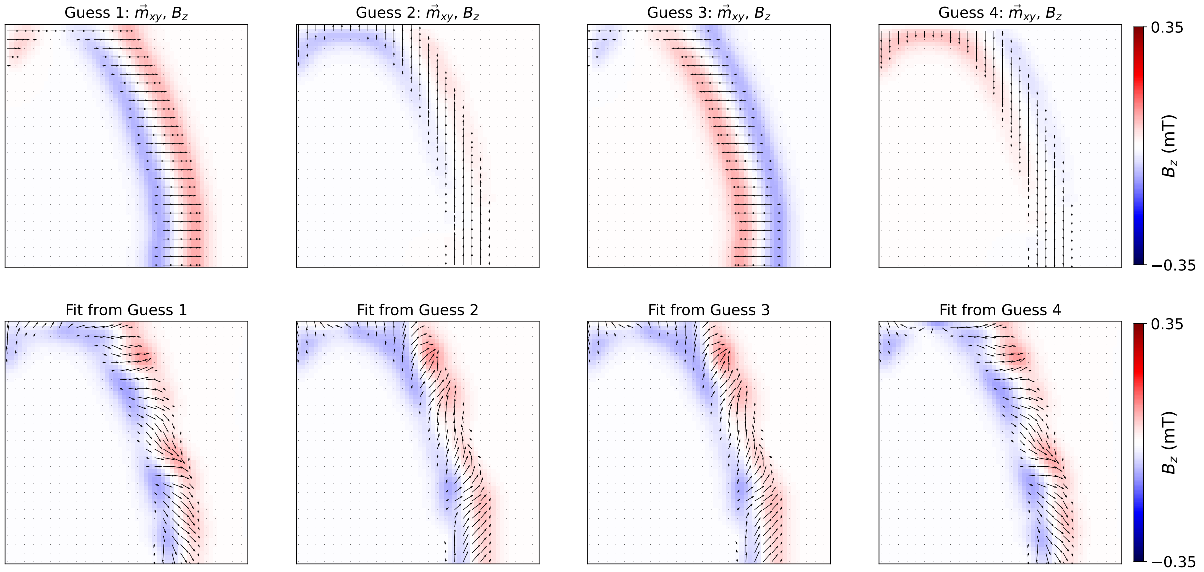

Figure S29: Fitting of domain wall with different initial conditions. Top row shows the different initial conditions. Bottom row shows the respective $\vec{m}_{xy}$ solution.

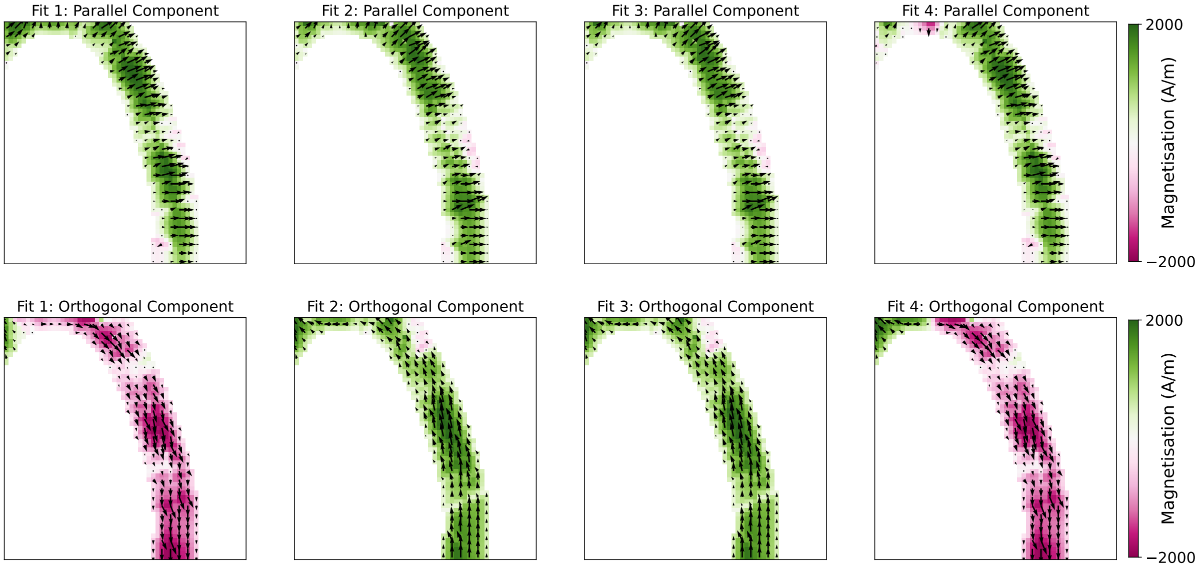

Figure S30: Degeneracy in $\vec{m}_{xy}$ solution. Decomposition of magnetisation fits from Fig.S29 into components parallel to and orthogonal to the domain wall. Arrows represent the magnetisation vector parallel to and orthogonal to the domain wall. Colors represent the polarity and magnitude of the parallel and orthogonal components. The components are retrieved by projecting the magnetisation vector of the solutions in Fig. S29 onto the domain wall vector given in Fig.S28(b).

S7.7 Additional Experimental Reconstruction Data

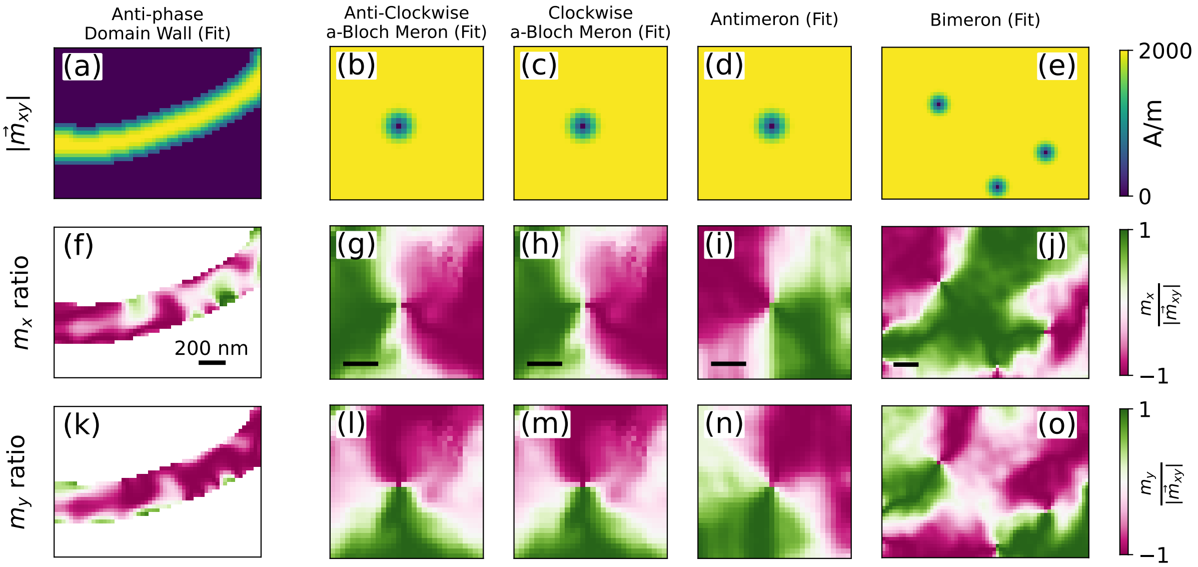

Figure S31: Additional information about the $\vec{m}_{xy}$ reconstruction of main text Fig 2. (a-e) Magnetisation norm and (f-j) ratio of mx to $|\vec{m}_{xy}|$, and (k-o) ratio of my to $|\vec{m}_{xy}|$. Columns along (a-e) correspond to the $\vec{m}_{xy}$ reconstruction of an antiphase domain wall, anti-clockwise and clockwise a-Bloch meron, antimeron and a bimeron, respectively

S8 Reversal Field Imaging

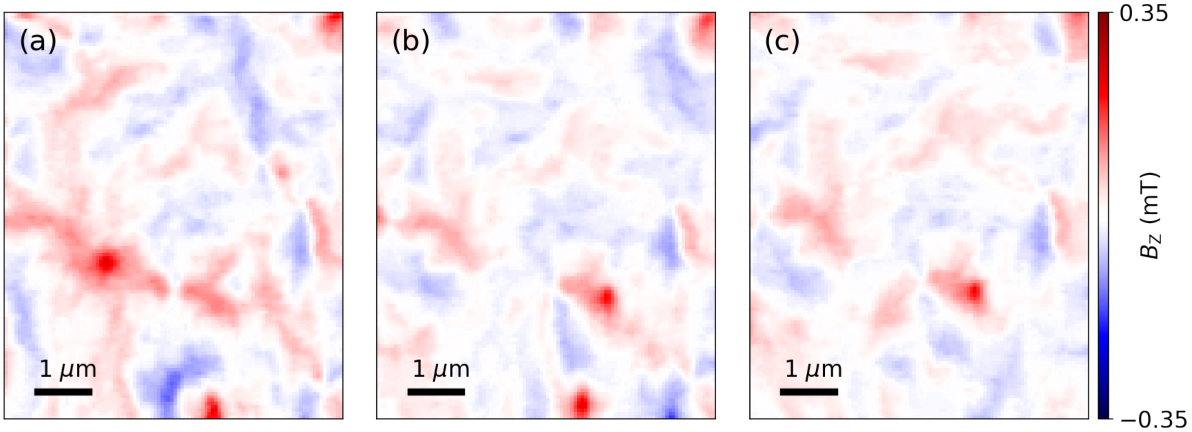

Figure S32: Reversal field imaging at remanence. Remanent state of α-Fe_2O_3 at room temperature after applying an *in-situ* in-plane magnetic field with magnitude varying from (a) 150mT, (b) 200mT and (c) 250mT.

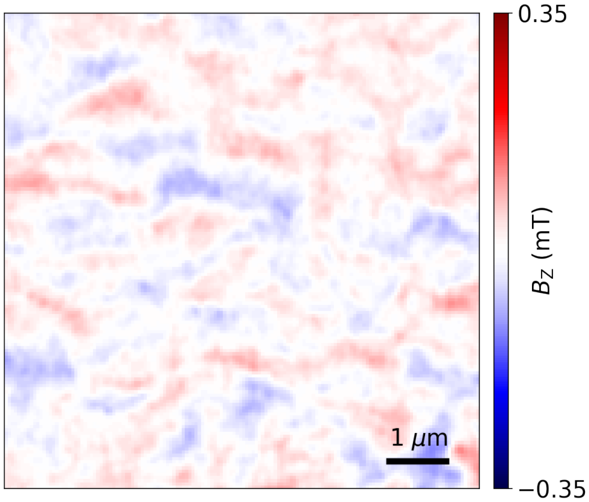

Figure S33: Annihilation of topological spin entities. Remanent state of α-Fe_2O_3 at room temperature after applying an *ex-situ* in-plane magnetic field of ~ 400 mT.

Upon application of strong in-plane magnetic fields, it is possible to drive AFM domain re-alignment which results in meron-antimeron annihilation (*1*). To confirm this, two permanent

magnet poles were aligned parallel to the sample surface and the scan x-axis resulting in a homogeneous field across the sample, variable via micrometer stages. Prior to the application of the external field, the sample was cooled down and warmed through the Morin transition, to reset and reinitialise the (anti-)meron distribution. The fields are then applied sequentially from low to high magnitude at room temperature. Since imaging under high off-NV-axis field is not possible, the external field was applied for a few minutes followed by NV imaging in zero field, see Fig. S32. Although the scans are approximately in the same area, open-loop scanning requires post processing, wherein the images have been registered using distinct field features in the scans. Several merons and antimerons are initially present. As the magnitude of the external field increases, annihilation processes occur, accompanied by an alteration of the surrounding trivial AFM textures. Due to the limited field magnitude that can be applied *in-situ*, large-scale annihilation of textures was driven by an *ex-situ* 400 mT field (*1*), see Fig. S33. This was achieved by two permanent magnet in close proximity producing a homogeneous field across the sample surface, whose direction (parallel to the *x*-axis) and magnitude were determined using a calibrated Hall sensor. Due to the probabilistic nature of the generation of topological textures via the Kibble-Zurek mechanism, the occurrence of merons with different projected vorticities (red vs blue cores) can vary across the sample (*17*). It is noted that while Fig. S33 shows predominately anti-clockwise a-Bloch merons (red cores), additional large area scans (see S9) confirm approximately similar distributions of merons with different projected vorticities (red vs blue cores) are obtained due to the absence of chiral interactions in α-Fe_2O_3 (*1*).

S9 Additional Data

The B_z maps in Fig. S34 show large range scans taken on different areas of the sample at room temperature. A small bias field is applied to infer the direction of the sample stray fields. Prior to imaging, the sample was thermally cycled through the Morin transition to reestablish the presence of merons and antimerons. Both maps show a distribution of clockwise and anti-clockwise a-Bloch merons without a clear bias towards either.

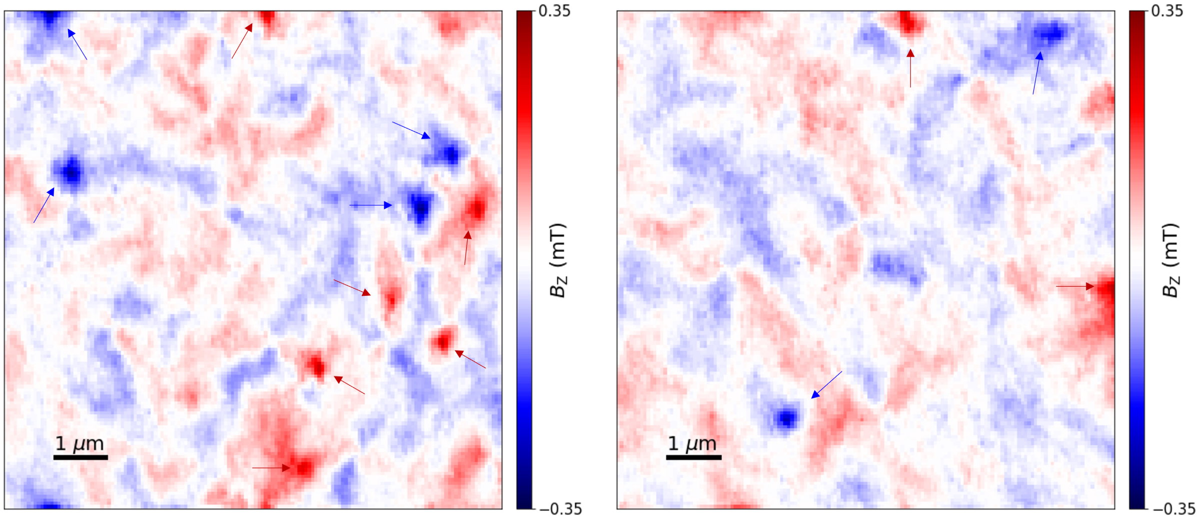

Figure S34: Large Area RT Images Taken After Thermal Cycling. Field imaging over a large area showing presence of both right and left a-Bloch merons indicated by blue and red arrows respectively.

S10 Dipolar Tensor

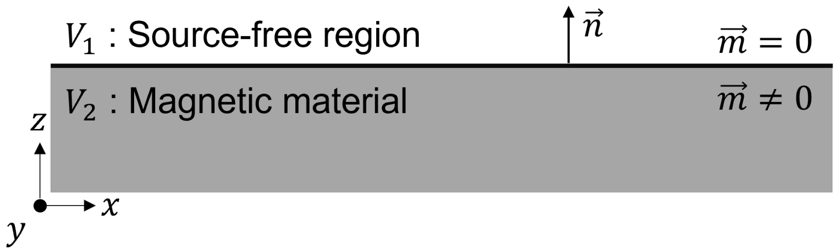

Figure S35: Illustration of a planar distribution of $\vec{m}$ in free space with two distinct volumes, V_1 and V_2, representing the source-free region and the magnetic material respectively.

The magnetic field analysis presented in section S4 including equation 1 of the main text, and the magnetisation reconstruction (S7), is built upon the dipolar tensor (eqn. S1) which maps a planar magnetisation $\vec{m}$ to a magnetic field distribution $\vec{B}$ at a given height d above it. Here, we show the derivation of the dipolar tensor.

In Figure S35, consider a planar magnetisation distribution of magnetisation $\vec{m}$, which is constant throughout z ($\frac{\partial}{\partial_z} m_z = 0$).

- In the source-free region, V_1, above the magnetisation distribution, Gauss's and Ampere's law hold for the magnetic field such that:

$$\vec{\nabla} \cdot \vec{B}_1 = \mu_0 \vec{\nabla} \cdot \vec{H}_1 = 0 \,.$$

Since $\vec{\nabla} \times \vec{H}_1 = 0$, a scalar potential, ϕ_1, can be defined as $\vec{H}_1 = -\vec{\nabla}\, \phi_1$ and therefore:

$$\mu_0 \vec{\nabla}^2\, \phi_1 = 0 \,.$$

Using $\mathcal{F}\{\frac{\partial}{\partial_x}\phi_1\} = ik_x\tilde{\phi}_1$, where $\tilde{\phi}_1 = \mathcal{F}\{\phi_1\}$ and $\mathcal{F}$ is the two-dimensional Fourier

transform along x and y,

$$\mathcal{F}\left\{\left(\frac{\partial^2}{\partial_x^2}+\frac{\partial^2}{\partial_y^2}+\frac{\partial^2}{\partial_z^2}\right)\phi_1(x,y,z)\right\}=\left(-k_x^2-k_y^2+\frac{\partial^2}{\partial_z^2}\right)\tilde{\phi}_1(k_x,k_y,z)=0\ . \tag{S30}$$

- In the magnetic material, V_2, $\vec{\nabla}\cdot\vec{B}_2=\mu_0\vec{\nabla}\cdot\left(\vec{H}_2+\vec{m}\right)=0$. Similarly, a scalar potential, ϕ_2, can be defined as $\vec{H}_2=-\vec{\nabla}\ \phi_2$ such that:

$$-\vec{\nabla}^2\cdot\phi_2+\vec{\nabla}\cdot\vec{m}=0\ .$$

In Fourier space:

$$\left(-k_x^2-k_y^2+\frac{\partial^2}{\partial_z^2}\right)\tilde{\phi}_2(k_x,k_y,z)=ik_xm_x+ik_ym_y+\underbrace{\frac{\partial}{\partial_z}m_z}_{=0}\ . \tag{S31}$$

The boundary conditions governing the perpendicular and horizontal components are (following from Gauss's and Ampere's law):

$$\left(\vec{B}_1-\vec{B}_2\right)\cdot\hat{n}=0\ , \tag{S32}$$

$$\left(\vec{H}_1-\vec{H}_2\right)\times\hat{n}=0\ , \tag{S33}$$

where $\hat{n}$ is the surface normal. Equation S30 has a solution of the form $\phi_1=c_1\mathrm{e}^{kz}+c_2\mathrm{e}^{-kz}$, where $k=\sqrt{k_x^2+k_y^2}$. Since $\lim_{z\to\infty}\phi_1=0$:

$$\phi_1=c_2\mathrm{e}^{-kz}\ . \tag{S34}$$

Similarly, equation S31 has a solution of the form $\phi_2=c_3\mathrm{e}^{kz}+c_4\mathrm{e}^{-kz}-\frac{\rho}{k^2}$, where $\rho=$

$ik_x m_x + ik_y m_y$. Since $\lim_{z\to-\infty} \phi_2 = 0$:

$$\phi_2 = c_4 \mathrm{e}^{kz} - \frac{\rho}{k^2} \; . \tag{S35}$$

Using the boundary condition in equation S33 and a = c_2, b = c_4,

$$\phi_1(k_x, k_y, z = 0) = \phi_2(k_x, k_y, z) \Longrightarrow a = b - \frac{\rho}{k^2} \; .$$

Using the boundary condition in equation S32,

$$\frac{\partial}{\partial_z}\phi_1\bigg|_{z=0} = \frac{\partial}{\partial_z}\phi_2\bigg|_{z=0} + m_z \; ,$$

$$\begin{aligned} \frac{\partial}{\partial_z}\phi_1 = -ka\mathrm{e}^{-kz} \Rightarrow \frac{\partial}{\partial_z}\phi_1(z=0) = -ka \; , \\ \frac{\partial}{\partial_z}\phi_2 = kb\mathrm{e}^{-kz} \Rightarrow \frac{\partial}{\partial_z}\phi_2(z=0) = kb \; . \end{aligned}$$

Then:

$$\begin{aligned} -ka &= kb + m_z \; , \\ a &= -b - \frac{m_z}{k} \; . \end{aligned} \tag{S36}$$

Substituting equation 7 in 8 and solving for a and b gives:

$$b = \frac{m_z}{2k} + \frac{\rho}{2k^2} \; , \tag{S37}$$

$$a = \frac{m_z}{2k} - \frac{\rho}{2k^2} \; . \tag{S38}$$

From equation S34, S37 and S38:

$$\phi_1(k_x, k_y, z) = \left(-\frac{ik_x m_x}{2k^2} - \frac{ik_y m_y}{2k^2} + \frac{m_z}{2k} \right) \mathrm{e}^{-kz} \,. \tag{S39}$$

Finally, using equation S39, the dipolar tensor is given as:

$$\begin{aligned} \vec{H}_1 &= -\vec{\nabla}\,\phi_1 \\ &= -\begin{bmatrix} \frac{k_x^2 m_x}{2k^2} & \frac{k_x k_y m_y}{2k^2} & \frac{ik_x m_z}{2k} \\ \frac{k_x k_y m_x}{2k^2} & \frac{k_y^2 m_y}{2k^2} & \frac{ik_y m_z}{2k} \\ \frac{ik_x m_x}{2k} & \frac{ik_y m_y}{2k} & -\frac{m_z}{2} \end{bmatrix} \mathrm{e}^{-kz} \\ &= -\frac{\mathrm{e}^{-kz}}{2k} \begin{bmatrix} \frac{k_x^2}{k} & \frac{k_x k_y}{k} & ik_x \\ \frac{k_x k_y}{k} & \frac{k_y^2}{k} & ik_y \\ ik_x & ik_y & -k \end{bmatrix} \begin{bmatrix} m_x \\ m_y \\ m_z \end{bmatrix} \,. \end{aligned}$$

References

1. Jani, H. *et al.* Antiferromagnetic half-skyrmions and bimerons at room temperature. *Nature* **590**, 74–79 (2021).

2. Jani, H. *et al.* Reversible hydrogen control of antiferromagnetic anisotropy in α-fe2o3. *Nature communications* **12**, 1–10 (2021).

3. Rondin, L. *et al.* Magnetometry with nitrogen-vacancy defects in diamond. *Reports on progress in physics* **77**, 056503 (2014).

4. Stefan, L. *et al.* Multiangle Reconstruction of Domain Morphology with All-Optical Diamond Magnetometry. *Physical Review Applied* **16**, 014054 (2021).

5. Hingant, T. *et al.* Measuring the Magnetic Moment Density in Patterned Ultrathin Ferromagnets with Submicrometer Resolution. *Physical Review Applied* **4**, 014003 (2015).

6. Harte, K. J. Theory of Magnetization Ripple in Ferromagnetic Films. *Journal of Applied Physics* **39**, 1503–1524 (1968).

7. van der Sar, T., Casola, F., Walsworth, R. & Yacoby, A. Nanometre-scale probing of spin waves using single electron spins. *Nature Communications* **6**, 7886 (2015).

8. Lima, E. A. & Weiss, B. P. Obtaining vector magnetic field maps from single-component measurements of geological samples. *Journal of Geophysical Research: Solid Earth* **114** (2009).

9. Dovzhenko, Y. *et al.* Magnetostatic twists in room-temperature skyrmions explored by nitrogen-vacancy center spin texture reconstruction. *Nature communications* **9**, 1–7 (2018).

10. Thiel, L. *et al.* Probing magnetism in 2d materials at the nanoscale with single-spin microscopy. *Science* **6926**, 1–7 (2019).

11. Blakely, R. J. *Transformations*, 311–358 (Cambridge University Press, 1995).

12. Wörnle, M. S. *et al.* Coexistence of Bloch and Néel walls in a collinear antiferromagnet. *Physical Review B* **103**, 094426 (2021).

13. Marchiori, E. *et al.* Nanoscale magnetic field imaging for 2d materials. *Nature Reviews Physics* **4**, 49–60 (2022).

14. Thoma, H. *et al.* Revealing the Absolute Direction of the Dzyaloshinskii-Moriya Interaction in Prototypical Weak Ferromagnets by Polarized Neutrons. *Physical Review X* **11**, 011060 (2021).

15. Dzyaloshinsky, I. A thermodynamic theory of "weak" ferromagnetism of antiferromagnetics. *Journal of Physics and Chemistry of Solids* **4**, 241–255 (1958).

16. Morrish, A. H. *Canted Antiferromagnetism: Hematite* (World Scientific, 1995).

17. Chmiel, F. P. *et al.* Observation of magnetic vortex pairs at room temperature in a planar α-Fe2O3/Co heterostructure. *Nature Materials* **17**, 581–585 (2018).

18. Besser, P. J., Morrish, A. H. & Searle, C. W. Magnetocrystalline anisotropy of pure and doped hematite. *Phys. Rev.* **153**, 632–640 (1967).

19. Litzius, K. *et al.* The role of temperature and drive current in skyrmion dynamics. *Nature Electronics* **3**, 30–36 (2020).

20. Galkina, E. G., Galkin, A. Y., Ivanov, B. A. & Nori, F. Magnetic vortex as a ground state for micron-scale antiferromagnetic samples. *Physical Review B - Condensed Matter and Materials Physics* **81**, 1–8 (2010).

21. Caron, J. *Model-based reconstruction of magnetisation distributions in nanostructures from electron optical phase images*. Ph.D. thesis, RWTH Aachen University (2017).
